# A brain-enriched circular RNA controls excitatory neurotransmission and restricts sensitivity to aversive stimuli

**DOI:** 10.1126/sciadv.adj8769

**Published:** 2024-05-24

**Authors:** Sebastian A. Giusti, Natalia S. Pino, Camila Pannunzio, Mora B. Ogando, Natalia G. Armando, Lillian Garrett, Annemarie Zimprich, Lore Becker, Maria L. Gimeno, Jeronimo Lukin, Florencia L. Merino, M. Belen Pardi, Olivia Pedroncini, Giuliana C. Di Mauro, Valerie Gailus Durner, Helmut Fuchs, Martin Hrabe de Angelis, Ines L. Patop, Christoph W. Turck, Jan M. Deussing, Daniela M. Vogt Weisenhorn, Olaf Jahn, Sebastian Kadener, Sabine M. Hölter, Nils Brose, Florian Giesert, Wolfgang Wurst, Antonia Marin-Burgin, Damian Refojo

**Affiliations:** ^1^ Instituto de Investigación en Biomedicina de Buenos Aires (IBioBA)–CONICET–Partner Institute of the Max Planck Society, Buenos Aires, Argentina.; ^2^ Molecular Neurobiology, Max Planck Institute of Psychiatry, Munich, Germany.; ^3^ Institute of Developmental Genetics, Helmholtz Zentrum München, German Research Center for Environmental Health, Munich, Germany.; ^4^ German Mouse Clinic, Helmholtz Zentrum München, Munich, Germany.; ^5^ Chair of Developmental Genetics, Munich School of Life Sciences Weihenstephan, Technical University of Munich, Freising, Germany.; ^6^ Institute of Experimental Genetics, Helmholtz Zentrum München, German Research Center for Environmental Health, Munich, Germany.; ^7^ Harvard Medical School, Boston, MA, USA.; ^8^ Department of Translational Research in Psychiatry, Max Planck Institute of Psychiatry, Munich, Germany.; ^9^ Molecular Neurogenetics, Max Planck Institute of Psychiatry, Munich, Germany.; ^10^ Department of Molecular Neurobiology, Max Planck Institute for Multidisciplinary Sciences, Göttingen, Germany.; ^11^ Department of Psychiatry and Psychotherapy, University Medical Center Göttingen, Georg-August-University, Göttingen, Germany.; ^12^ Biology Department, Brandeis University, Waltham, MA, USA.; ^13^ Munich Cluster of Systems Neurology (SyNergy), Munich, Germany.; ^14^ German Center for Neurodegenerative Diseases (DZNE) Site Munich, Munich, Germany.

## Abstract

Circular RNAs (circRNAs) are a large class of noncoding RNAs. Despite the identification of thousands of circular transcripts, the biological significance of most of them remains unexplored, partly because of the lack of effective methods for generating loss-of-function animal models. In this study, we focused on circTulp4, an abundant circRNA derived from the *Tulp4* gene that is enriched in the brain and synaptic compartments. By creating a circTulp4-deficient mouse model, in which we mutated the splice acceptor site responsible for generating circTulp4 without affecting the linear mRNA or protein levels, we were able to conduct a comprehensive phenotypic analysis. Our results demonstrate that circTulp4 is critical in regulating neuronal and brain physiology, modulating the strength of excitatory neurotransmission and sensitivity to aversive stimuli. This study provides evidence that circRNAs can regulate biologically relevant functions in neurons, with modulatory effects at multiple levels of the phenotype, establishing a proof of principle for the regulatory role of circRNAs in neural processes.

## Teaser

Brain-enriched circRNA circTulp4 modulates brain function and aversive stimulus sensitivity in mice.

## INTRODUCTION

Exonic circular RNAs (circRNAs) are a large class of noncoding RNAs with unique features (*1*). They are generated by an alternative splicing mechanism called backsplicing, wherein a donor site splices with an upstream acceptor site, resulting in a covalently closed, single-stranded RNA molecule. Because of their circular structure, circRNAs exhibit a natural resistance to exonucleases, and their average half-lives are reported to be three to five times longer than those of linear RNAs (*2*, *3*). Although they were first described over 30 years ago (*4*), circRNAs were long believed to be rare, nonfunctional artifacts of RNA processing. However, with the advent of high-throughput RNA sequencing (RNA-seq) technology and circRNA-specific bioinformatics (*5*–*7*), thousands of circRNAs have been identified in various eukaryotic taxa, revealing their abundance and dynamic expression and suggesting their functional relevance. Some circRNAs have been shown to function as microRNA (miRNA) sponges (*8*, *9*) or interact with specific proteins modulating their activity (*10*) or subcellular localization (*11*). In addition, a small number of circular transcripts have been reported to act as templates for the translation of peptides in a cap-independent manner (*12*–*14*). However, recent statistical analyses of RNA-seq data suggest that many circRNAs, especially those expressed at low levels, may be nonfunctional products of splicing errors (*15*). Therefore, the functional relevance of each identified circRNA remains largely unknown and requires further investigation.

In our previous work, we conducted a large-scale identification of circular transcripts and their linear isoforms from human and mouse central nervous system (CNS) samples using RNA-seq and ad hoc bioinformatic analyses (*16*). We found hundreds of circRNAs that exhibited higher expression levels than their linear isoforms, consistent with previous studies indicating that circRNAs are enriched in nervous tissue (*17*). Further, we examined samples from primary mouse neuronal cultures and observed a differential expression of circRNAs during neuronal maturation, with a global increase of circular transcripts in mature neurons. We also conducted subcellular fractionation of brain samples, followed by RNA-seq, and showed that many circRNAs are abundant in synaptic fractions. Together, these findings suggest a fascinating potential role for circRNAs as regulators of neurotransmission or synaptic plasticity phenomena (*18*).

Recent studies have demonstrated that many circRNAs exhibit age-dependent accumulation in brain tissues, independent of the linear RNA expression of their host genes (*19*). In addition, the dysregulation of circular transcripts has been implicated in neurological disorders such as Alzheimer’s disease (*20*) and Parkinson’s disease (*21*), further suggesting relevant involvement of circRNAs in brain function.

Previous studies have used short hairpin RNAs (shRNAs) directed against the backsplicing junction to down-regulate circRNAs and investigate their role in specific brain structures or neurons in culture (*22*–*24*). However, these approaches are limited by potential off-target effects associated with shRNA-mediated knockdown. Moreover, even with alternative technologies such as CRISPR-CasRx (*25*, *26*), targeting specific brain areas may hinder comprehensive phenotyping analyses.

To date, only a small fraction of circRNAs have been functionally characterized in vivo. In *Drosophila*, depletion of the highly abundant circMbl reduced viability and impaired locomotion and flight (*27*), while circMbl knockdown in the CNS led to alterations in synaptic vesicle dynamics (*28*). In mammals, CDR1as/ciRS-7 is the most abundant and the best-characterized circRNA. Notably, it differs from most circRNAs because it is derived from a sequence with no annotated host gene, and the prevailing assumption has been that it lacks a linear isoform derived from the same locus. It was later shown, however, that its sequence is embedded in mature noncoding linear transcripts expressed at low levels (*29*). This atypical feature allowed the development of the first knockout (KO) mouse model generated by a classical disruption of the gene locus (*30*). CDR1as KO mice showed miRNA dysregulation in the brain and alterations in sensorimotor gating (*30*). Moreover, electrophysiological measurements in CDR1as KO single (autaptic) hippocampal neurons in culture revealed dysfunctional synaptic transmission. However, CDR1as is an exceptional circRNA since it does not have a linear mRNA counterpart derived from the same locus and has the unique property of containing more than 70 binding sites for miR-7, an miRNA with critical functions during CNS development. Therefore, for any typical circRNA derived from a protein-coding gene, the field still lacks a mammalian in vivo model for circRNA loss of function that allows a neuron and brain-oriented, functional characterization based on a comprehensive and multilevel analysis of the phenotype. This highlights the unresolved question of the actual relevance of circRNAs in the brain.

To address this open question, we selected a circRNA derived from the *Tulp4* gene (circTulp4) from a previous RNA-seq screening (*16*) based on its high abundance in the brain. We confirmed that circTulp4 is enriched in the brain and synaptic fractions, and its levels increase during neuronal maturation. To assess the biological significance of circTulp4 in vivo, we developed a circTulp4-deficient (CD) mouse model by targeting the splice acceptor (SA) site of the backsplicing reaction. This resulted in a marked reduction in circRNA levels without affecting the levels of linear tubby like protein 4 (Tulp4) mRNA or Tulp4 protein. Using a comprehensive neurobiological phenotyping platform covering more than 40 traits, we demonstrate that circTulp4 deficiency leads to heightened sensitivity to sensory and environmental stress stimuli accompanied by deficits in excitatory neurotransmission. Our findings highlight the importance of circTulp4 in regulating brain function and suggest a broader role for circRNAs in neuronal activity and stress response.

## RESULTS

### CircTulp4 is enriched in the brain and synaptic fractions and regulates synaptic transmission in primary neurons

**Fig. 1.**
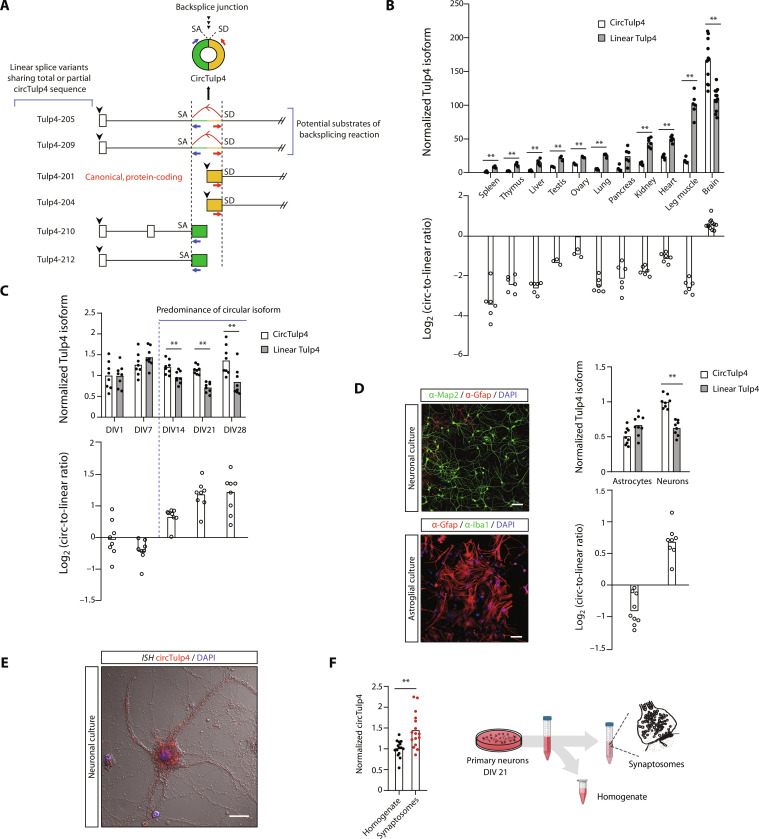
CircTulp4 is the primary transcriptional output of the *Tulp4* gene in the brain and is enriched in synaptic fractions. (**A**) CircTulp4 and linear splice variants. In each transcript, boxes represent exonic sequences, and lines represent intronic sequences. The red and blue arrows represent primers that are used to detect circTulp4 by reverse transcription quantitative polymerase chain reaction (RT-qPCR). “SA” and “SD” are the splice acceptor and splice donor sites, respectively, involved in the backsplicing reaction. (**B**, **C**, **D**, and **F**) Relative quantification of circTulp4 and linear Tulp4 by RT-qPCR. All values were normalized with the average of two housekeepers from the same sample: RPL19 and TATA-binding protein. (B) CircTulp4 and linear Tulp4 in different mouse tissues. The average value for normalized circTulp4 in the spleen was arbitrarily set in “1” (*n* = 12 for the brain, *n* = 3 for testis and ovary, and *n* = 6 for other organs; mixed-effects analysis with multiple comparison tests). (C) CircTulp4 and linear Tulp4 in primary mouse neurons at different maturation time points (DIV). The average value for normalized circTulp4 at DIV1 was arbitrarily set in 1 [*n* = 8 independent cultures followed from DIV1 to DIV28; two-way repeated-measures analysis of variance (RM ANOVA) and Sidak’s multiple comparison test]. (D) Contrary to mature primary neurons (DIV21), there are no significant differences between circTulp4 and linear Tulp4 levels in mouse primary astroglial samples (*n* = 8 independent neuronal cultures and *n* = 9 independent astroglial cultures; two-way ANOVA and Sidak’s multiple comparison test). Left: Representative images of immunofluorescence analysis confirming the specific cell type enrichment of neuronal and astroglial cultures. Scale bars, 100 μm. (**E**) Representative image of fluorescent in situ hybridization with probes for circTulp4 showing its somatodendritic distribution in a DIV21 neuronal culture. Scale bar, 20 μm. (F) circTulp4 is enriched in synaptic fractions of DIV21 neurons (*n* = 15 homogenate-synaptosome independent pairs; paired *t* test). In all cases, columns represent mean values. **P < 0.01.

For functional characterization, we selected circTulp4 (fig. S1), a human-conserved, 1946-nucleotide-long exonic circRNA identified in a previous study (*16*). CircTulp4 derives from the *Tulp4* gene (ENSMUSG00000034377), which produces 12 mRNA splice variants. Six of them share sequences with circTulp4, which are exonic for four of them and intronic for the other two (Fig. 1A, the transcription start site for each transcript is indicated by a black arrowhead). Tulp4-201 (ENSMUST00000039655.3) is the canonical transcript of the gene and encodes for the main protein isoform that has a still uncharacterized function in mammals. Tulp4-209 (ENSMUST00000149756.8) also codes a noncanonical, less abundant protein isoform. The sum of both Tulp4-201 and Tulp4-209, the most abundant linear isoforms, are referred to as “linear Tulp4.” CircTulp4 presumably arises from backsplicing the same pre-mRNA that can be spliced into transcripts Tulp4-205 and Tulp4-209 since these are the only *Tulp4* transcripts containing the complete circTulp4 sequence.

Comparing the abundance of circular and linear Tulp4 transcripts in different mouse organs, we found that circTulp4 levels are highest in the brain, the only organ in which circTulp4 is the main transcriptional output of the gene (Fig. 1B). In primary forebrain neuronal cultures, the predominance of circTulp4 over its linear counterparts progressively increases during neuronal maturation (Fig. 1C and fig. S2A). It is established after 14 days of in vitro culture (DIV14), a period characterized by the development of dendritic spines and the peak of synaptogenesis (*31*, *32*). By analyzing samples derived from primary neuronal and astrocytic cultures, we found that the circular-to-linear Tulp4 predominance is present in neurons but not in astrocytes (Fig. 1D and fig. S2B). In neuronal cultures, microtubule-associated protein 2 was used as a neuronal marker, and glial fibrillary acidic protein (Gfap) was used to detect potential astrocyte contamination; in astroglial cultures, Gfap was used as an astrocytic marker, and ionized calcium-binding adapter molecule 1 was used to detect potential microglial contamination (Fig. 1D, left). In mature cultured neurons, we confirmed by in situ hybridization with a probe directed to the backsplicing junction that circTulp4 is present in somatic and axodendritic compartments (Fig. 1E). By performing subcellular fractionation of mature neuronal cultures, we found that circTulp4 is enriched in synaptic fractions (Fig. 1F and fig. S2C). Changes in circTulp4 expression throughout neuronal development could arise from a concomitant increase in synaptic activity. To test a potential relationship between circTulp4 expression and localization with synaptic activity, we stimulated primary forebrain neurons with 4-aminopyridine/bicuculline for 3 hours. CircTulp4 levels were remarkably stable (fig. S2, D and E), suggesting that orthogonal mechanisms or other homeostatic processes regulate its expression and localization.

**Fig. 2.**
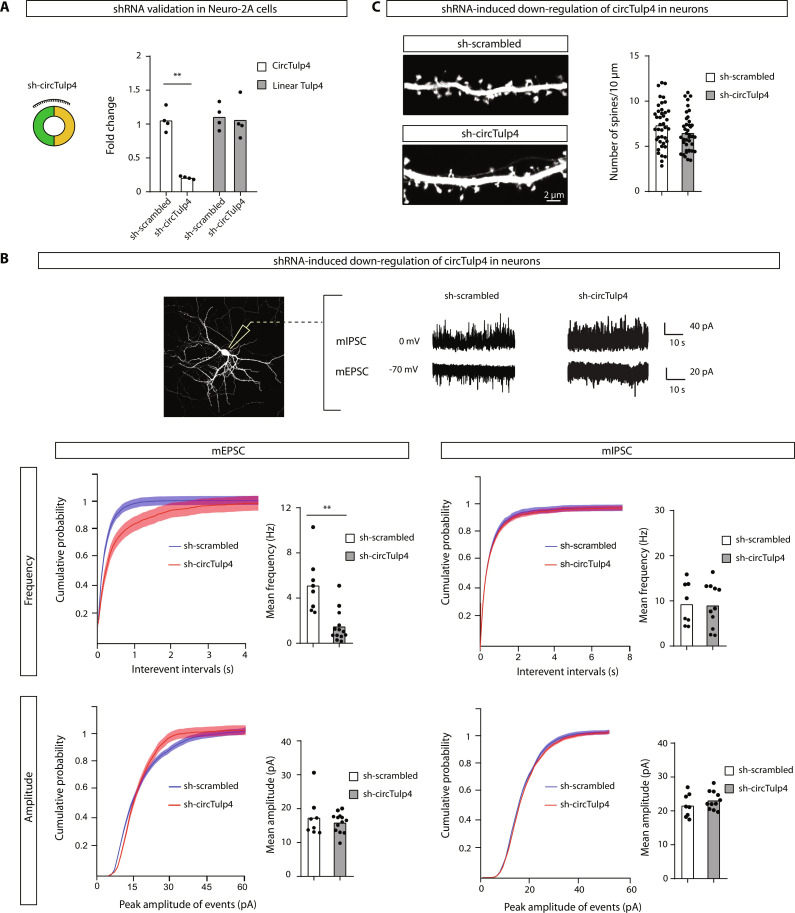
CircTulp4 regulates the frequency of mEPSCs in primary neurons. (**A**) Loss-of-function experiments were performed with an shRNA vector that selectively down-regulates circTulp4 without affecting the linear isoform (sh-circTulp4). A scrambled shRNA sequence (sh-scrambled) was used under the control condition. The efficiency and specificity of the shRNAs were tested in Neuro-2A cells (two-way RM ANOVA and Sidak’s multiple comparison test, *n* = 4). (**B** and **C**) Mouse primary hippocampal neurons were cotransfected at DIV12 to DIV13 with red fluorescent protein and either sh-scrambled or sh-circTulp4. Dendritic spine analysis and electrophysiological recordings of transfected neurons were performed at DIV21 to DIV25. (B) Top: Representative sample traces. Bottom: The frequencies of mEPSCs (sh-scrambled, *n* = 8 cells; sh-circTulp4, *n* = 13 cells) measured by whole-cell recordings were strongly diminished when circTulp4 was negatively regulated, whereas miniature inhibitory postsynaptic currents (mIPSCs; sh-scrambled, *n* = 8 cells; sh-circTulp4, *n* = 11 cells) were not altered (*t* test with Welch’s correction). Recorded cells were from two independent primary cultures. (C) CircTulp4 down-regulation did not affect the density of dendritic spines (*t* test; *n* = spine density of 40 dendritic segments per condition, from four independent primary cultures). In all cases, columns represent mean values. **P < 0.01.

To begin the exploration of the consequences of circTulp4 loss of function, we designed and validated an shRNA construct directed to the backsplice junction of circTulp4 (sh-circTulp4) that down-regulates circTulp4 levels without affecting its linear counterpart (Fig. 2A). Mature hippocampal primary neurons transfected with this construct displayed a reduction in the frequency of miniature excitatory postsynaptic currents (mEPSCs) compared to the scrambled control (sh-scrambled) transfected cells (Fig. 2B). Notably, mEPSCs were not affected when linear Tulp4 was down-regulated (fig. S3, A and B). Furthermore, the cotransfection of a circTulp4-overexpressing construct restores the reduction in mEPSCs’ frequency induced by the sh-circTulp4, demonstrating that the effect of the shRNA is specific (fig. S3, C and D). In contrast, no effects were observed in the miniature inhibitory postsynaptic currents (mIPSCs) (Fig. 2B) or the density of dendritic spines (Fig. 2C) after circTulp4 down-regulation, suggesting that circTulp4 might exert functional effects specifically onto excitatory synapses.

### Mutation of the splicing acceptor down-regulates circTulp4 levels in vivo

CircRNA biogenesis is promoted by the formation of base pairing of inverted repeats located on both sides of the exon(s) undergoing backsplicing [reviewed in (*33*)]. These reverse complementary matches (RCMs) facilitate circularization by bringing adjacent introns into proximity and stabilizing their interactions. Here, we used the BLAST algorithm to comprehensively analyze the presence of RCMs within the introns flanking circTulp4. Our investigation revealed numerous RCMs (listed in the data S1), with a substantial proportion of these overlaps coinciding with short interspersed nuclear elements (fig. S4). On the basis of the length and percentage of complementarity, we identified five RCMs as promising candidates for mediating circularization (fig. S4).

However, the genetic removal of these intronic sequences to generate an in vivo model for circTulp4 loss of function poses a substantial risk. Determining which is the primary contributor to circularization or assessing whether these sequences serve redundant roles remains challenging. Such an approach could potentially lead to unintended consequences, including the inadvertent removal of critical RNA binding protein binding sites and potential alterations to the nascent transcript’s three-dimensional structure, affecting the locus’ global regulation.

**Fig. 3.**
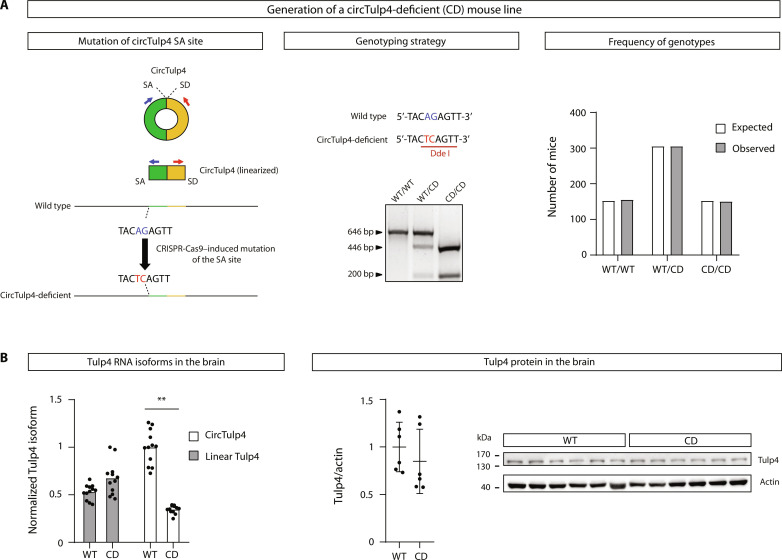
A mouse model for circTulp4 loss of function. (**A**) Left: Diagram depicting circTulp4 genomic DNA sequence and the splicing sites involved in its biogenesis. CRISPR-Cas9–mediated mutagenesis of AG to TC disrupts the SA site in the CD allele. The red and blue arrows represent primers that are used to detect circTulp4 by RT-qPCR. Middle: Representative genotyping based on a PCR–restriction fragment length polymorphism (RFLP) strategy. Genotyping primers amplify a 646–base pair (bp) fragment of *Tulp4*; consecutive digestion with the Dde I restriction enzyme allows distinguishing between the different genotypes by RFLPs. WT/WT, 646 bp; WT/CD, 646 bp + 446 bp +200 bp; CD/CD, 446 bp + 200 bp. WT, wild type. Right: CircTulp4 mice offspring follows the expected Mendelian ratios (*n* = 610 mice from 90 heterozygous breedings; chi-square test, *P* = 0.979). (**B**) Left: In the brain of CD mice, circTulp4 levels, measured by relative quantification by RT-qPCR, are diminished, on average, at 65.4%, whereas linear Tulp4 levels are not different between genotypes. All values were normalized with the average of two housekeepers from the same sample: RPL19 and TATA-binding protein. The average value for normalized circTulp4 in WT mice was arbitrarily set in 1 (*n* = 12 mice per genotype; two-way ANOVA and Tukey’s multiple comparison test). Right: Tulp4 protein levels in the brain are not altered in CD mice. Western blot with Tulp4 antibody (*n* = 6 mice per genotype; *t* test with Welch’s correction). In (B), columns represent mean values. **P < 0.01.

Instead, to characterize the potential functions of circTulp4 in vivo, we used a CRISPR-Cas9–based strategy to generate a CD mouse model based on the specific depletion of the circular isoform of *Tulp4* gene through direct mutation of the canonical SA site involved in the backsplicing reaction (Fig. 3A, left). Our approach takes advantage of the fact that the SA site of circTulp4 is not used in the maturation of the most abundant linear Tulp4 transcript, which encodes for the canonical protein (Fig. 1A). Therefore, the designed mutation should not affect the linear mRNA abundance.

As expected, CD mice have a specific down-regulation of circTulp4 levels in different organs (fig. S5B). In the brain, the organ with the highest circTulp4 content, we observed a down-regulation of 65% compared to wild-type littermates, whereas linear Tulp4 and Tulp4 protein levels do not differ between genotypes (Fig. 3B and fig. S5D). We determined by Sanger sequencing that the circTulp4 molecules detected in CD mice are produced by the juxtaposed, next downstream 5′-AG-3′ acting as a cryptic SA site for the backsplicing reaction (fig. S5A), with less efficiency than the wild-type SA site.

The frequency of homozygous wild-type and CD mice from heterozygous breedings do not differ from the expected Mendelian ratios (Fig. 3A, right), demonstrating the lack of a sublethal effect of circTulp4 down-regulation. Notably, female CD mice have increased body weight compared to wild-type littermates, whereas the body weight of males does not differ between genotypes (fig. S6A). Besides, CD mice have no overt anomaly in the brain’s gross anatomy (fig. S6B) and no differences in the number or morphology of astrocytes and microglia (fig. S6C).

### CD mice display higher sensitivity to aversive stimuli

We performed an in-depth characterization to assess the impact of circTulp4 down-regulation on neurological traits and behavior (fig. S7A). Only male mice were used for the behavioral phenotyping since the increased body weight observed in CD females (fig. S6A) and the arguably causal or secondary defects in metabolism might indeed be a potential confounder effect on the behavioral tests.

The SHIRPA screening performed in the first steps of the behavioral characterization indicated that CD mice have normal reflexes (pinna and startle reflexes and contact righting), posture (tail elevation, trunk curl, and gait), and autonomous (urination and defection) functions (fig. S7B). Other neurological traits, such as tremors and limb grasping, were absent in CD mice, and biting or vocalizations were equal between genotypes. Other behavioral aspects such as touch escape, transfer arousal, and body position remained unchanged in CD mice. Similarly, the down-regulation of circTulp4 did not affect sensorimotor gating (fig. S7C), hearing sensitivity (fig. S7D), muscle function (fig. S7E), or motor coordination (fig. S7F). Similarly, short-term memory (fig. S7H) and social behavior (fig. S7G) were also preserved in CD mice.

**Fig. 4.**
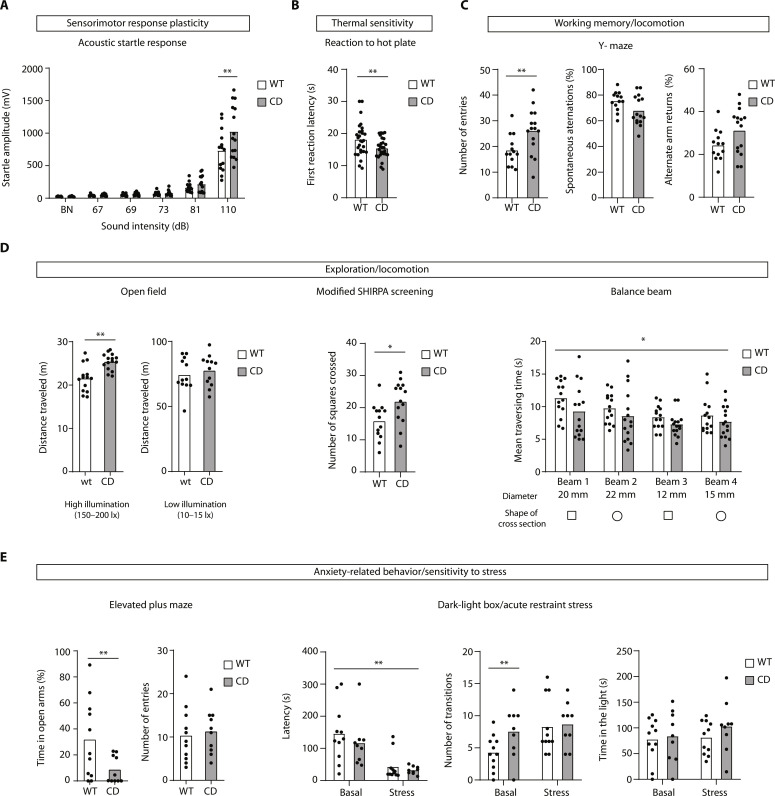
CD mice have an increased sensitivity to stressful stimuli. (**A**) Startle response elicited by acoustic stimuli. Background noise (BN) intensity was 65 dB. CD mice display increased reactivity at 110 dB (two-way RM ANOVA and Bonferroni’s multiple comparisons test). (**B**) CD mice have an increased thermal sensitivity measured in the hot plate test, with faster reaction times in the first response (*t* test with Welch’s correction). (**C**) CD mice display increased arm entries compared to control littermates in the Y-maze. In addition, there is a tendency for a reduced percentage of spontaneous alternations (*P* = 0.0519) and an increased percentage of alternate arm returns (*P* = 0.0564) (*t* test with Welch’s correction). (**D**) CD mice show hyperactivity in novel environments under specific conditions. Left: The hyperactivity is observed in the OF arena under high-illumination conditions but not under dim illumination (*t* test). Middle: Hyperactive behavior was also evidenced in the modified SHIRPA screening (*t* test). Right: In the balance beam test, CD mice showed reduced traversing times on all beams (two-way RM ANOVA). (**E**) CD mice display increased sensitivity to stressful conditions. Left: CD mice display a reduced percentage of time spent in the open arms in the elevated plus maze (*t* test with Welch’s correction). Right: Dark-light box before (basal) and after acute restraint stress. Hyperlocomotion of CD mice is observed in the high number of transitions under basal conditions, which are also increased in WT mice after stress. Stress diminishes the latency to enter the lit compartment in both genotypes (two-way RM ANOVA and Sidak’s multiple comparisons test). In all cases, columns represent mean values. *P < 0.05 and **P < 0.01. Three batches of mice were used, and the “*n*” of each experiment depends on the batch used in that test (see details in fig. S7).

CD mice show increased startle reactivity to acoustic stimuli (Fig. 4A) and higher thermal sensitivity (Fig. 4B), suggesting an elevated arousal state. Moreover, CD mice displayed hyperlocomotion reflected in the increased number of entries in the Y-maze (Fig. 4C), the high traveling distance in the bright-illuminated open field (OF) and the SHIRPA locomotion assessment, and in the reduced traversing times in the balance beam test (Fig. 4D).

However, hyperlocomotion is not a pervasive and spontaneous feature of CD mice behavior, as demonstrated by its absence in the OF under low-illumination conditions (Fig. 4D), which also allowed us to distinguish it from exploratory behavior. Hyperlocomotion in CD mice is triggered under specific and potentially stressful conditions, suggesting a high-anxiety phenotype with increased arousal. These results prompted us to explore the emotional-related behavior of CD mice more closely.

We did not find a consistent phenotype of generalized high anxiety but specific differences. In the elevated plus maze test, CD mice spent less time than wild-type controls in the open arms (Fig. 4E, left); however, no such differences were observed in other anxiety-related behavioral tests such as the marble burying test (fig. S7I, middle) or in the time spent in the center of the OF (fig. S7I, left). Besides, we found no alterations in helplessness, an endophenotype of depression, measured by the forced swim test (fig. S7I, right).

We next performed the dark-light test before and after an acute restraint to assess whether stress had a differential influence on anxiety levels. No genotype differences were observed in the latency or time spent in the lit compartment during the dark-light box test (Fig. 4E, right). Acute stress, nonetheless, induced a significant reduction in latency times in both genotypes. We also observed, under basal conditions, an increased number of transitions in CD mice compatible with the previously observed hyperlocomotion. Notably, stress increased the number of transitions of wild-type mice, mimicking the hyperlocomotive phenotype of CD mice (Fig. 4E, right). This fact further suggests that the increase in locomotion is a response of aroused mice under stressful conditions. Overall, we interpret the hyperreactivity of CD mice to a sudden loud sound or unbearable heat and the hyperlocomotion displayed under specific aversive contexts (e.g., bright light in novel environments) as indications of higher sensitivity to aversive stimuli.

### CircTulp4 depletion provokes presynaptic alterations in excitatory neurotransmission

**Fig. 5.**
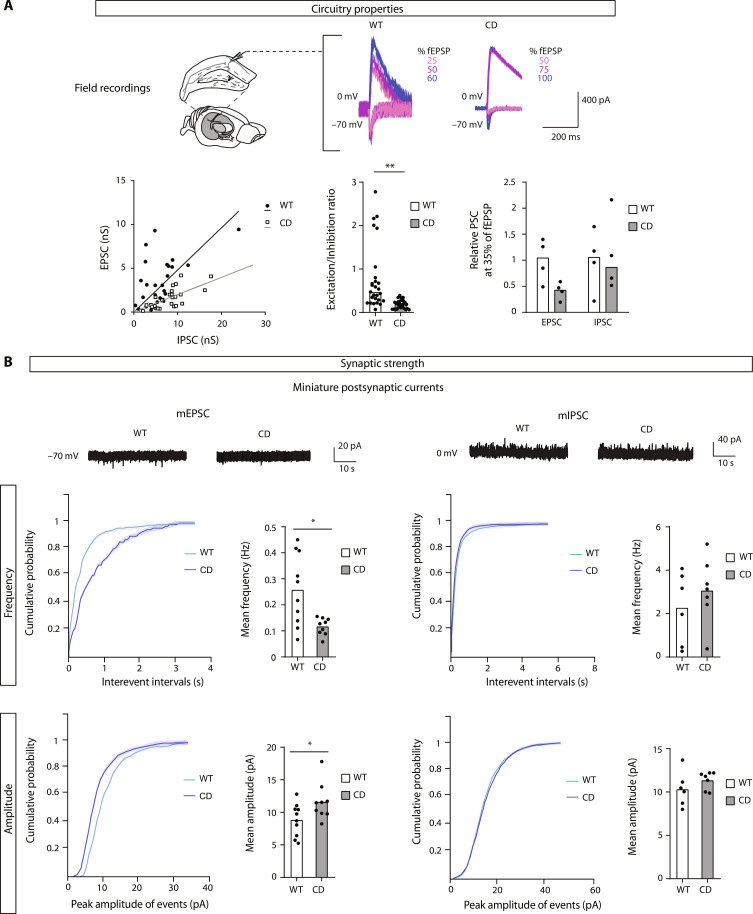
Electrophysiological consequences of circTulp4 loss of function. (**A**) Circuitry properties. Left: E/I balance is strongly diminished in CD mice across different stimulation intensities. Slope (95% confidence interval): 0.3514 to 0.6095 for WT (*n* = 7 cells from three mice); 0.1609 to 0.2344 for CD (*n* = 9 cells from three mice). Middle: Mean of E/I ratio from different stimulation intensities (*t* test; *n* = 27 per genotype). Right: EPSC and IPSC at 35% of field excitatory postsynaptic potential (fEPSP), showing that a decrease in the excitatory currents explains the decrease in E/I balance [bars represent median values, Mann-Whitney test, *n* = 4 cells for WT and 3 cells for CD mice, *P* = 0.057 (EPSC, WT versus CD)]. Top: Representative sample traces. (**B**) CA1 neurons were evaluated in hippocampal slices of 6-week-old mice by patch clamp recordings. The frequencies of mEPSCs (WT, *n* = 9 cells from three mice; CD, *n* = 10 cells from three mice) are strongly diminished, while mIPSCs (WT, *n* = 6 cells from two mice; CD, *n* = 7 cells from two mice) are not altered (*t* test with Welch’s correction). A moderate increase in the amplitude of the mEPSCs in CD mice was also observed. Top: Representative sample traces. Unless otherwise stated, columns represent mean values. *P < 0.05 and **P < 0.01.

To explore the impact of circTulp4 loss of function on synaptic transmission in vivo, we conducted an electrophysiological characterization of CD mice. First, we assessed neurotransmission at the microcircuitry level in acute brain slices containing the hippocampus. To do so, we stimulated the Schaffer collateral axons and measured evoked monosynaptic EPSCs and IPSCs in CA1 pyramidal neurons. We observed a reduction of the evoked excitation/inhibition (E/I) balance in CD mice across different stimulation intensities that are explained by a decrease in the excitatory currents (Fig. 5A). Consistent with this observation, our findings revealed lower frequencies of mEPSCs at the synaptic level, similar to those observed in primary neurons transfected with shRNA against circTulp4. In contrast, no alterations were detected in mIPSCs (Fig. 5B) or the input resistance (fig. S8A, left). We also observed a mild shift of the action potential threshold to more negative membrane potentials in CD mice (fig. S8A, right), which we interpreted as a compensatory mechanism.

**Fig. 6.**
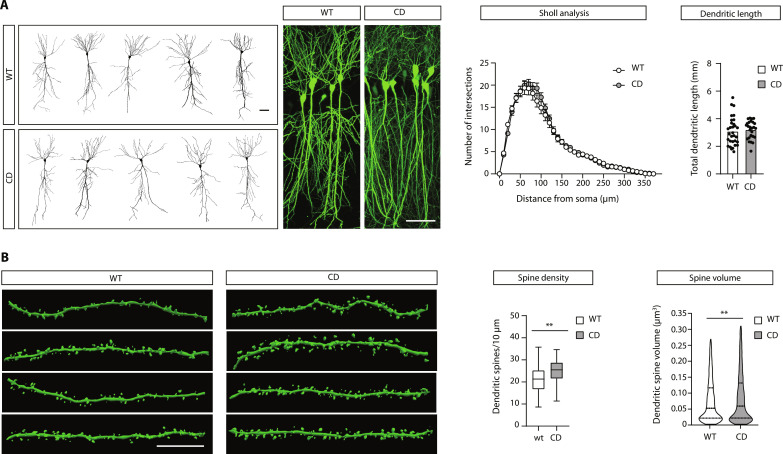
Morphological analysis of CA1 neurons. A subset of neurons was genetically labeled using the Thy1EGFP mouse line. (**A**) Representative tracings (left) and confocal stack images (right) of CA1 neurons of Thy1EGFP–wild-type (control) and Thy1EGFP-CD mice. Scale bars, 50 μm. No differences were found between genotypes in the total dendritic length (*t* test with Welch’s correction) or the complexity of the dendritic arborization estimated by a Sholl analysis (two-way RM ANOVA; WT, *n* = 31 neurons from five mice; CD, *n* = 27 neurons from six mice). (**B**) Representative digital reconstructions of dendritic segments with dendritic spines. Scale bar, 10 μm. The average spine density in CD mice was 17% (95% confidence interval, 30 to 3%) higher than wild-type mice (*t* test with Welch’s correction; data shown as boxes with Tukey whiskers). Spine volume in CD mice was 13% (95% confidence interval, 22 to 4%) higher than wild type (Mann-Whitney test; data shown as violin plots). Spine density and volumes were calculated from *n* = 99 dendrites from six mice for WT and *n* = 98 dendrites from six mice for CD. **P < 0.01.

We hypothesized that a reduction in the frequency of mEPSCs could be explained by a reduction in the density (or volume) of dendritic spines, which are thought to be a structural correlate of the number (or strength) of excitatory synapses. To test this hypothesis, we conducted a morphological analysis of CA1 pyramidal neurons, assessing the total dendritic length, the complexity of the dendritic arborization, and the density and volume of dendritic spines (Fig. 6). To do so, a subset of neurons was genetically labeled with enhanced green fluorescent protein (EGFP) by crossing CD mice with the Thy1EGFP mouse line. Unexpectedly, we found a moderate increase in the density and volume of dendritic spines (Fig. 6A and fig. S8B), which we interpreted as a compensatory mechanism for the reduced strength of excitatory neurotransmission.

**Fig. 7.**
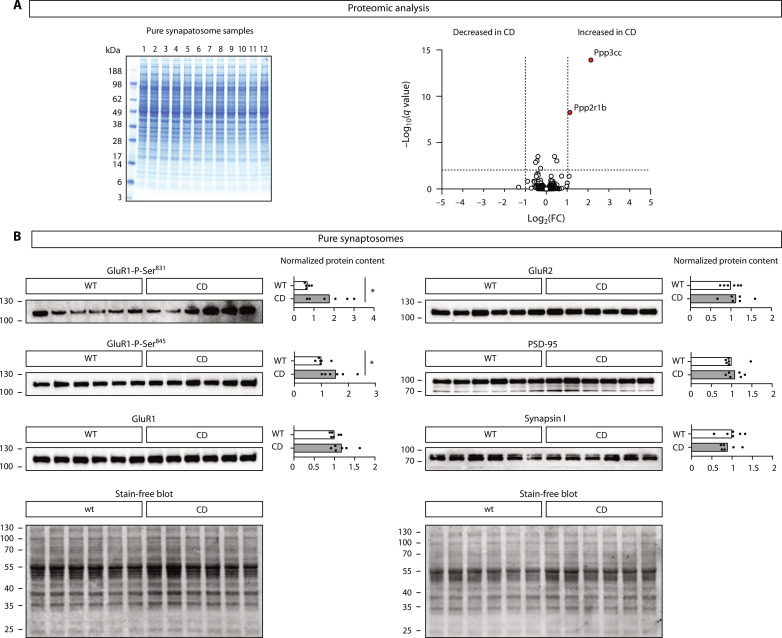
Quantitative proteomic analysis of synaptic fractions. (**A**) Mass spectrometry (MS)–based, label-free synaptosomal protein quantification. Left: Gel electrophoresis (SDS-PAGE) followed by colloidal Coomassie staining shows homogeneous protein content and consistent band patterns among synaptosomal protein samples. Right: Volcano plot of proteins identified in pure synaptosomes from CD or wild-type forebrains. Proteins with a fold change (FC) of >2 or <0.5 and *q* values of <0.01 were considered as significantly dysregulated in CD mice. Top hits are indicated in red circles (*n* = 6 pure synaptosomal samples per genotype, each obtained from the forebrain of one mouse). (**B**) Western blot of pure synaptosomes shows dysregulation in the phosphorylation status of GluR1 (*t* test with Welch’s correction, *n* = 6 samples per genotype).

To test whether the reduced frequency of mEPSCs in CD mice was explained by an alteration in the levels of synaptic proteins, we conducted a quantitative proteomic analysis. Pure synaptosomal fractions were prepared from wild-type and CD mice. Coomassie blue staining of SDS–polyacrylamide gel electrophoresis (PAGE) of the samples showed overall homogeneous protein profiles (Fig. 7A, left). Of 1821 proteins identified in the synaptosomal samples (listed in data S2), only two were above the fold change and the significance cutoff thresholds but failed the validation by Western blot (Fig. 7A, right, and fig. S8C). Therefore, we concluded that the protein composition of the synaptosomal compartment is preserved in CD mice.

Taking into account that AMPA receptors (AMPARs) mediate mEPSCs, we explored the glutamate receptor 1 (GluR1) and GluR2 AMPAR subunits in more detail. In agreement with the proteomic data, we observed by Western blot in an independent batch of pure synaptosomal samples that GluR1 and GluR2 levels were not changed between genotypes (Fig. 7B). As an additional control, we confirmed that PSD-95 and Synapsin-1, as postsynaptic and presynaptic markers, respectively, were not altered in those samples, which also reinforces our previous observations that CD mice do not exhibit structural alterations at synapses. We found, however, alterations in the phosphorylation status of GluR1 AMPAR subunits, which further suggests a compensatory mechanism rather than a molecular explanation for the reduced frequency of mEPSCs.

Since the number of synapses was not diminished, we reasoned that the decreased frequency of mEPSCs could be explained by a reduction in the release probability from presynaptic terminals or an increased presence of silent synapses, namely, synapses mediated exclusively by *N*-methyl-d-aspartate (NMDA) receptors, with no detectable contribution from AMPARs at the postsynapse. Along this line, we next asked whether decreasing circTulp4 only in the postsynaptic compartment might be sufficient to reproduce the diminishment in the frequency of mEPSCs. To address this point, we down-regulated circTulp4 in a subset of sparse CA1 neurons without affecting circTulp4 levels in the afferent CA3 neurons. Hippocampal neural precursors of embryonic day 15.5 (E15.5) wild-type embryos were transfected by in utero electroporation with a plasmid expressing mRFP (volume marker) with either an sh-circTulp4 construct or an sh-scrambled control plasmid (fig. S9A). This approach allowed us to diminish circTulp4 levels exclusively in the postsynaptic neuron.

Intrinsic properties were not different between the two treatments (differences between treatments expressed as means ± SEM; input resistance: 96.25 ± 103.1 megohms, *t* test, *P* = 0.386; action potential threshold: 5.96 ± 4.52 mV, *t* test, *P* = 0.245). We also found no differences in the density of dendritic spines between sh-circTulp4– and control-transfected neurons (fig. S9B), further suggesting that the morphological changes observed in CD mice neurons are compensatory.

**Fig. 8.**
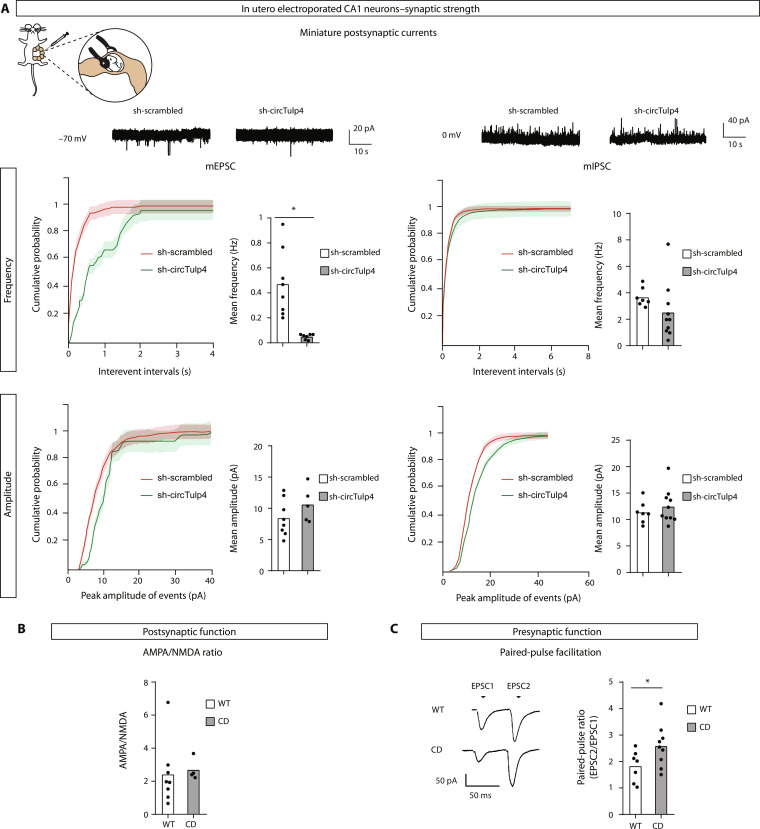
The presynaptic function is affected in CD mice. (**A**) Hippocampal neural precursors of wild-type CD1 embryos were in utero electroporated at E15.5 with sh-circTulp4. Sh-scrambled was used under the control condition. Under both conditions, mRFP was cotransfected to identify transfected neurons. Six weeks after birth, transfected CA1 neurons were evaluated in hippocampal slices of 6-week-old mice by patch-clamp recordings (see also fig. S9A). The frequencies of mEPSCs (sh-scrambled, *n* = 8 cells from two mice; sh-circTulp4, *n* = 7 cells from two mice) are strongly diminished, while mIPSCs (sh-scrambled, *n* = 7 cells from two mice; sh-circTulp4, *n* = 10 cells from two mice) are not altered (*t* test with Welch’s correction). No effects were observed in the amplitude of mEPSCs or mIPSCs. Top: Representative sample traces. (**B**) No changes between genotypes were observed in the postsynaptic component of synaptic strength estimated by the AMPA/NMDA ratio (*t* test with Welch’s correction; WT, *n* = 8 cells from two mice; CD, *n* = 4 cells from two mice). (**C**) The presynaptic function was explored with the PPF protocol. The ratio of EPSC2/EPSC1 was recorded at intervals of 50 ms. CD mice showed a higher paired-pulse ratio (*t* test with Welch’s correction; WT, *n* = 7 cells from three mice; CD, *n* = 9 cells from two mice). In all cases, columns represent mean values. *P < 0.05.

Miniature postsynaptic currents and the dendritic spine density of transfected CA1 neurons were analyzed 6 weeks after birth. Notably, similar to what was observed in CD mice, the down-regulation of circTulp4 in transfected postsynaptic neurons led to a reduction in the frequency of mEPSCs (Fig. 8A), showing that it is sufficient to reduce circTulp4 levels in the postsynaptic neuron to reduce the number of EPSCs. This finding suggested two possible, although not mutually exclusive, explanations: CircTulp4 depletion in the postsynapse can increase the proportion of silent synapses or reduce the presynaptic probability of release by retrograde synaptic messengers.

Considering that the strength of the synapse is established by the abundance of AMPARs at the surface of excitatory synapses, we hypothesized that the reduced frequency of mEPSCs in CD mice could be explained by a reduction in the level of GluR1 or GluR2 anchored at the postsynaptic density (PSD). Since total levels of GluR1 and GluR2 are not different between genotypes in the pure synaptosomal fractions (Fig. 7, A and B), we speculated that a higher proportion of those proteins could be localized outside of the PSD either in lateral synaptic membranes or retained in recycling endosomes at expenses of the AMPAR content in the PSD of CD synapses. To test this hypothesis, we further fractionated synaptosomal fractions into synaptic plasma membranes (SPMs), containing all synaptic membrane proteins but excluding the synaptosome’s endocytic compartment, and the derived PSD2T fraction, which only contains proteins anchored into the PSD. We found no differences in the levels of GluR1 or GluR2 in SPM or PSD2T fractions (fig. S9C), indicating that these proteins were not differentially retained in endosomes or synaptic membranes lateral to the PSD, respectively. Therefore, we conclude that the proportion of surface, PSD-anchored AMPARs is not altered in CD mouse synapses.

Nonetheless, normal global levels of AMPARs in isolated PSD do not entirely exclude the potential existence of an increased number of silent synapses in CD mice since these unchanged total levels of AMPARs quantified in tissue samples might be unevenly distributed among individual active and silent synapses. To assess the proportion of silent synapses, we electrophysiologically measured the ratio between AMPA- and NMDA-mediated currents in whole-cell recordings of hippocampal CA1 neurons and found no differences between genotypes (Fig. 8B). This indicated that CD mice do not have a higher proportion of silent synapses compared to wild-type littermates.

Because we could not find evidence for the reduction in the number of surface AMPARs or the proportion of silent synapses, we reasoned that the reduced frequency of mEPSCs should be explained by a presynaptic alteration in CD mice. Therefore, we performed a paired-pulse facilitation (PPF) protocol at the Schaffer collateral-CA1 synapses to check the presynaptic function. PPF is a form of short-term, activity-dependent synaptic plasticity common to most chemically transmitting synapses, manifested as an enhancement in the amplitude of the second of two rapidly evoked EPSCs. We found an increase in the PPF ratio (EPSC2/EPSC1) in CD synapses (Fig. 8C), explained by a reduced EPSC1 in CD mice, which is interpreted as a reduction in the basal probability of neurotransmitter release in those synapses. Together, these results show that circTulp4 regulates the probability of release of synaptic vesicles in excitatory synapses and, in turn, the E/I balance of brain circuits.

### CircTulp4 is unlikely to work by sponging miRNAs or being a template for protein translation

Which are the molecular bases that mediate circTulp4 biological functions? We focused on two previously demonstrated mechanisms for a few other circRNAs. First, we aimed to test whether circTulp4 is a decoy target for miRNAs. Second, we explored the potential translation of a peptide encoded in a circTulp4-specific open reading frame (ORF).

**Fig. 9.**
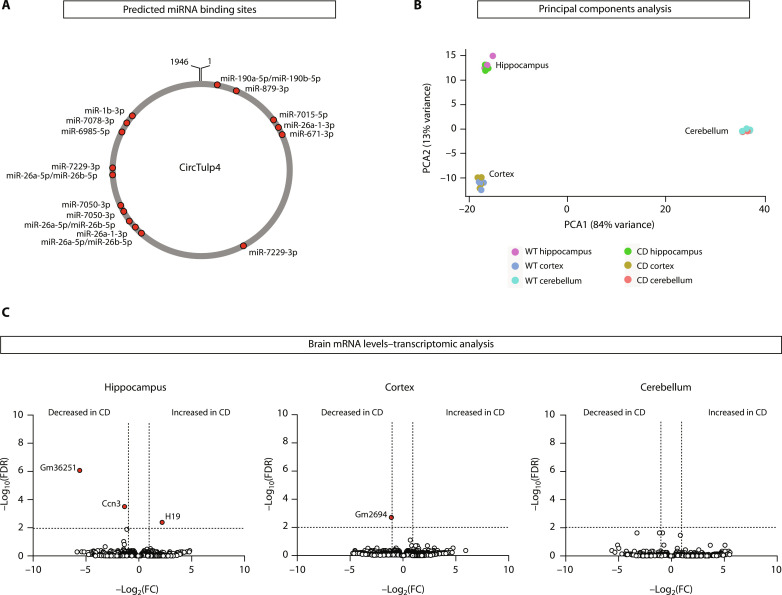
CircTulp4 does not function as a sponge for miRNAs. (**A**) Bioinformatically predicted miRNA binding sites in the circTulp4 sequence. Only target sites with a score of 70 or higher are shown (see also fig. S10A). (**B** and **C**) Transcriptomic analysis of hippocampus, cortex, and cerebellum of wild-type and CD mice (hippocampus: WT, *n* = 2; CD, *n* = 3; cortex: WT, *n* = 3; CD, *n* = 3; cerebellum: WT, *n* = 3; CD, *n* = 2). (B) Principal components analysis (PCA) shows a differential transcriptional landscape for each brain region but no differences between genotypes. (C) Volcano plots representing differential gene expression between wild-type and CD mouse brain samples. mRNAs with a fold change of >2 or <0.5 and false discovery rate (FDR) of <0.01 were considered as significantly dysregulated in CD mice (see also fig. S10B). Top hits are indicated in red circles.

We performed a bioinformatic screening for miRNA binding sites in circTulp4 with the target prediction database miRDB (*34*) and found 16 binding sites for 13 different miRNAs (Fig. 9A and fig. S10A). We hypothesized that a biologically relevant interaction of circTulp4 with miRNAs would lead to a dysregulation of their mRNA targets. We experimentally assessed the potential impact of circTulp4 down-regulation in the brain by performing a comparative transcriptomic analysis of three brain regions between wild-type and CD mice. We found differences in the transcriptomic landscape of the different brain regions (Fig. 9B), but only marginal differences appeared between genotypes (Fig. 9C). Of ~14.700 transcripts in each brain region, three were found dysregulated in the hippocampus, one in the cortex and none in the cerebellum. None of those transcripts is a target for the miRNAs with predicted binding sites in circTulp4 (fig. S10B). In summary, these results suggest that circTulp4 does not function as an miRNA sponge in the brain and, more generally, does not alter the transcriptional landscape.

**Fig. 10.**
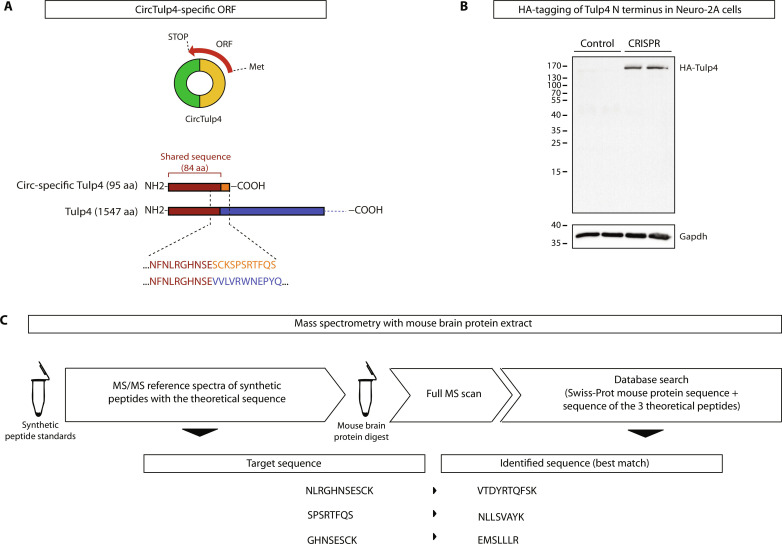
CircTulp4 is not a template for translating peptides. (**A**) Top: CircTulp4 encodes an ORF spanning the backsplice junction. Bottom: Homology at protein level between the theoretical circular-specific Tulp4 peptide [95 amino acids (aa)] and the Tulp4 canonical protein (1547 amino acids). (**B**) Neuro-2A cells were transfected with a Cas9-expressing plasmid, a single guide RNA–expressing plasmid, and a donor vector that, upon homology-directed recombination, introduces an HA-tag to the N terminus of Tulp4. Western blot analysis with an α-HA-tag antibody of protein extracts from control and CRISPR transfected (CRISPR) shows that HA-Tulp4, but not HA–circular-specific Tulp4, is detectable. (**C**) MS analysis reveals that brain samples do not contain the theoretical peptide circular-specific Tulp4. First, the synthetic theoretical peptides (NLRGHNSESCK, GHNSESCK, and SPSRTFQS, 1 pmol each) were analyzed to collect MS/MS reference spectra. Then, a mouse brain protein digest (1 μg) was injected into the MS using a parallel reaction monitoring (PRM) mode with an inclusion list for the theoretical peptides following a full MS scan. No exact matches were found (see also fig. S10C).

Considering that it has been shown that some circRNAs can be translated, we bioinformatically explored the sequence of circTulp4 in search of circular-specific ORFs. In particular, we found an ORF spanning the backsplice junction, potentially encoding a 95 amino acid–peptide, referred to as “circular-specific Tulp4.” Notably, the predicted peptide is identical (amino acids 1 to 84) to the N terminus of the Tulp4 protein (Fig. 10A). To test whether circular-specific Tulp4 exists in cells, we used CRISPR in Neuro-2A cells to genetically add the hemagglutinin (HA) tag to the N terminus shared by Tulp4 and circular-specific Tulp4. We could detect HA-Tulp4 (~170 kDa) by Western blot, demonstrating that the genetic modification was achieved, but we found no signal compatible with circular-specific Tulp4 (~11 kDa) in the same samples (Fig. 10B). This result, however, did not exclude the possibility that circular-specific Tulp4 is present in mature neurons or brain tissue. Therefore, we used mass spectrometry (MS) to search for circular-specific Tulp4 directly in the mouse brain proteome. Three synthetic peptides were used as standards to increase the assay’s sensitivity. Those three peptides are the expected products of trypsin digestion of circular-specific Tulp4, which also contain the amino acids that allow the discrimination from the Tulp4 N terminus. None of the three peptides were identified in mouse brain extracts. Instead, in each case, the best match was an alternative peptide with a similar mass but a different fragmentation pattern (Fig. 10C and fig. S10C). Together, our results suggest that circTulp4 is not a template for translating a circular-specific peptide in the brain.

## DISCUSSION

In this study, we have successfully generated a circRNA-deficient mouse model and performed a comprehensive functional analysis of a canonical brain–enriched circRNA, circTulp4. Our findings demonstrate that circTulp4 regulates excitatory neurotransmission and influences behavior, highlighting the functional relevance of circRNAs in the brain.

Previous studies have used shRNAs that target the backsplicing junction to reduce circRNA expression and explore their functions within distinct brain regions (*24*, *35*). However, it is worth noting that the selectivity of this approach toward specific brain areas limits a thorough characterization of phenotypic outcomes. Therefore, a fundamental limitation in the field has been the lack of mammalian loss-of-function models for typical circRNAs that allow for comprehensive, multilevel functional characterization. This is mainly due to the technical challenges associated with disrupting the expression of circRNAs. Two potential strategies are to mutate the splice donor (SD) or SA sites or delete entire introns. The first strategy is impractical, as the circular and linear isoforms usually share the splicing sites, so their mutation will affect splicing and, ultimately, the expression of both isoforms. The second option is technically challenging as introns are large and complex to remove during homology-directed recombination. However, it is also risky, as intronic sequences often contain enhancers and other transcriptional or epigenetic regulatory regions (*36*, *37*) or even encode for miRNAs (*38*) or other noncoding RNAs (*39*), adding a severe confounding factor in the constructive (or etiological) validity of an animal model. A variation of that strategy, entailing some of the mentioned limitations, is removing inverted repeats situated on either side of the exon(s) subject to backsplicing, which has occasionally been used in mouse (*40*) and invertebrate models (*41*). Last, even if these limitations could be overcome, many circRNAs influence the expression of or are influenced by their linear mRNA counterparts, so reducing or suppressing the expression of one isoform could ultimately affect the expression of the other. Of course, since many of these regulatory regions are cell- or tissue-specific, there is no way to test them in a reliable in vitro system. Arguably, all these technical barriers have, so far, discouraged scientists from developing these mouse models.

To date, CDR1as KO is the only reported genetically modified mouse line for a brain-enriched circRNA. This mouse line was generated by a large deletion of the CDR1as genomic locus based on the rationale, valid for this isolated case, that there is little, if any, expression of its linear isoform or any unrelated noncoding RNA (*8*, *9*, *30*).

We realized that because of the structure of the *Tulp4* locus, the SA site of circTulp4 is not involved in synthesizing the linear Tulp4 transcript that encodes for the Tulp4 protein. Therefore, we envisioned that its mutation would lead only to the specific down-regulation of the circRNA variant. Thus, we used the CRISPR-Cas9 technique to specifically mutate, via homology-directed repair, the SA site involved in the backsplicing reaction. This mutation led to a 65% reduction in the efficiency of circTulp4 generation without affecting the linear counterpart.

CircTulp4 has been previously reported by other groups (*17*) and us (*16*), as one of the most abundant circRNAs in brain samples based on RNA-seq data. We have shown that circTulp4 is most highly expressed in the brain, the only organ in which the circular isoform is the main transcriptional output of the Tulp4 gene. With neuronal maturation, the circular/lineal ratio increases (actually becomes inverted). Cell type– and maturation stage–dependent variations of the circular-to-linear ratio have also been reported for other transcripts (*16*, *42*). Since circRNAs synthesis is cotranscriptional, circularization and splicing may compete against each other (*10*), although there is some evidence that backsplicing of circRNAs can also occur posttranscriptionally (*43*). In addition, cross-talk between linear (or its translated protein) and circular forms influencing transcription in regulatory feedback loops has been described too (*27*, *44*). However, in Tulp4 CD mice, Tulp4 mRNA levels remain constant, suggesting a regulated mechanism that specifically increases circTulp4 levels along maturation and in brain tissue, independently of the mRNA synthesis.

We found that circTulp4 is enriched in synaptic fractions, and we proved, with independent methods, i.e., genetically modified mice and shRNAs in wild-type neurons, that circTulp4 controls the strength of excitatory neurotransmission without affecting the levels of synaptic proteins. Moreover, circTulp4 regulates the probability of release of synaptic vesicles in excitatory synapses and, in turn, the E/I balance of brain circuits. Homeostatic mechanisms acting in a rectifying direction are observed in CD mice, i.e., a slight increase in the density of dendritic spines and hyperphosphorylation of GluR1. Although frequent in constitutive genetically modified animals, these homeostatic mechanisms could not fully compensate for the functional consequences of circTulp4 down-regulation. This fact further underlines the critical role of circTulp4 in neuronal and brain physiology, which also affects behavior, regulating the arousal state and fine-tuning the sensitivity to aversive stimuli.

The limited experimental validation of the biological functions of circRNAs is coextensive to their molecular mechanisms of action. Nonetheless, circRNAs have been reported to act as decoys, endogenous competitors (sponges) for miRNAs and proteins, and to serve as scaffolds for circRNA-protein complexes. For example, the mouse circSry, associated with testis development (*45*), contains 16 binding sites for miR-138 (*8*). It has also been suggested that sponging of miR-124 by circHIPK3 can regulate cell growth in human cells (*46*). From this class, CDR1as, abundantly expressed in mammalian brains, is the best-characterized example, with more than 70 conserved binding sites for miR-7. Reduced CDR1as expression in human cell lines led to reduced levels of mRNAs harboring binding sites for miR-7, suggesting that CDR1as functions as a sponge for this miRNA (*8*, *9*). The CDR1as–miR-7 axis is also modulated by Cyrano, a long noncoding RNA that binds miR-7 and triggers target-directed miRNA degradation (*47*). Unexpectedly, in CDR1as KO mice, miR-7 levels are reduced, and, in contrast with the cell line model, miR-7 targets are up-regulated (*30*). This discrepancy further emphasizes the value of an in vivo model to gain insight into the potential biological roles of circRNAs. On the other hand, some circRNAs, including circZNF609 and circMbl, were found to have a natural internal ribosomal entry site and to undergo translation (*8*, *9*). However, whether circRNA-encoded peptides are functional remains unclear. Here, we have shown that circTulp4 is not a decoy target for miRNAs based on RNA-seq data from different brain regions and bioinformatic analysis of potential miRNA binding sites in the circTulp4 sequence. In line with our findings, circTulp4 was not among the eight circRNAs that were found to be associated with AGO2HITS-CLIP data from two regions of the human brain (*48*). Besides, our results demonstrate that circTulp4 is not functioning as a template for translating peptides. Moreover, the conserved levels of synaptic proteins suggest that circTulp4 does not regulate local synaptic translation. Additional studies are needed to determine the molecular mechanisms that mediate circTulp4 cellular and physiological effects. Identifying molecular interactors of circTulp4 [experimental strategies are reviewed in (*49*)] will help narrow down the potentially relevant molecular pathways and generate a new set of hypotheses to unravel the underlying molecular mechanisms in the future.

In conclusion, this study provides compelling evidence that a canonical circRNA, circTulp4, has a biologically relevant cell-intrinsic regulatory function in neurons. Specifically, our findings demonstrate that circTulp4 influences neurotransmitter release at excitatory synapses and exacerbates responses to aversive and anxiogenic stimuli in vivo. Hence, these findings provide significant proof of principle for the broader potential functional relevance of circRNAs in the brain and pave the way for future investigations into their precise mechanisms of action and downstream effects on neural circuits and behavior.

## MATERIALS AND METHODS

### Mice

Mice were group-housed (up to four mice per cage, whenever possible) under standard laboratory conditions (22° ± 1°C, 55 ± 5% humidity) with a 12:12-hour light:dark schedule with food and water ad libitum. For staging of embryos, noon on the day of the appearance of a vaginal plug was treated as E0.5, and the day of birth was considered postnatal day 0 (P0). E16.5 embryos from the CD1 strain were used to prepare dissociated neuronal cultures, and P2 pups were used to prepare astroglial cultures. Timed pregnant E15.5 CD1 mice were used for in utero electroporation experiments.

CD mouse line was kept on a C57BL/6 breeding background. In some cases, CD mice were crossed with the Thy1EGFP mouse line (*50*). For genotyping, DNA was isolated from the ear tissue by alkaline lysis, and polymerase chain reaction (PCR) was performed under standard conditions using primers listed in table S1. In the case of CD mice, genotyping PCR was followed by digestion with Dde I restriction enzyme to distinguish wild type from CD alleles. All animal experiments for this study were approved by the appropriate local authorities. Animal studies performed at IBioBA-CONICET were done in accordance with local regulations and the National Research Council Guide for the Care and Use of Laboratory Animals, followed at IBioBA-CONICET and approved by the local Institutional Animal Care and Use Committee. At the German Mouse Clinic (GMC), animals were maintained in individually ventilated cages cages with water and standard mouse chow according to the directive 2010/63/EU, German national laws, and GMC housing conditions (www.mouseclinic.de). All tests performed were approved by the responsible authority of the district government of Upper Bavaria. Animal protocol numbers are the following: primary cultures, 2020-02-NE; in utero electroporation, 2020-05-NE; mouse generation, ROB 55.2-2532.Vet 02-14-205, 46-16; behavioral characterization at GMC, ROB 55.2-1-54-2532-46-2016; behavioral characterization at IBioBA-CONICET, 2020-06-NE.

### Bioinformatic mapping of RCMs

To identify RCMs in the introns flanking circTulp4, we used the BLAST algorithm to find matches between one intron and the reverse complement of the other. We implemented this into our shRNAtools R package (https://github.com/ipatop/shRNAtools). We used the function *find_rcm*. This function takes as input the coordinates of flanking introns and the annotation version and extracts the sequence of each intron and performs blast. It returns a table with the RCMs found and a txt file with the full blast alignment for each RCM.

### Generation of CD mice via CRISPR-Cas9–mediated mutagenesis

CRISPR target sites were identified using a bioinformatic tool developed by the Zhang laboratory at Massachusetts Institute of Technology as previously described (*51*). Alternative target sequences (gRNA_SA_circTulp4#1 and gRNA_SA_circTulp4#2; see table S1) were selected according to their quality score and cloned as complementary oligonucleotides into the BbsI sites of a pbs-U6-chimaericRNA plasmid (*52*). Plasmid-encoded target sequences were cotransfected in Neuro-2A cells with a Cas9-expressing vector (*53*) to test the Cas9 cutting efficiency at the locus of interest in the mouse genome.

For pronuclear microinjections, crRNA, tracrRNA (both from Integrated DNA Technologies Inc.), and a high-performance liquid chromatography (HPLC)–purified single-stranded oligodeoxynucleotide (ssODN) repair template (Metabion) were used. The ssODN (ssODN_SA_circTulp4; see table S1) was designed to replace the canonical splicing acceptor site AG by TC. One additional mutation was added in the protospacer adjacent motif site to avoid ssODN recognition and cleavage by Cas9. The three oligos were diluted in T10E0.1 injection buffer (10 mM tris-HCl and 0.1 mM EDTA), filtrated through a centrifugal filter (Ultrafree, polytetrafluoroethylene, Millipore), and stored in single-use aliquots at −80°C. On the day of injection, a thawed aliquot containing crRNA/tracrRNA heteroduplex (0.6 pmol/μl), ssODN (50 ng/μl), and Cas9 mRNA (25 ng/μl) was added Cas9 protein to a final concentration of 50 ng/μl. For microinjections, zygotes were obtained by mating C57BL/6 males with superovulated C57BL/6 females (Charles River). Zygotes were injected into one pronucleus and transferred into pseudo-pregnant CD1 female mice to obtain live pups. Seven of 14 pups born presented indels, and one male pup happened to be a mosaic founder. The founder male was bred to wild-type C57BL/6 female mice for germline transmission. Mutant allele transmission was confirmed by genotyping PCR, followed by digestion with Dde I restriction enzyme and DNA sequencing.

### Dissociated mouse neuron and astroglial cultures

Hippocampal and cortical neurons were prepared from CD1 mouse embryos (E17.5 to E19.5) and maintained in Neurobasal-A medium with 2% B27 supplement and 0.5 mM GlutaMAX (all from Gibco) at 37°C and 5% CO_2_. Neurons were plated on Poly-D-Lysine (PDL)-coated or PDL- and laminin-coated plates or glass coverslips at the desired cell density (5 × 10^4^ cells per well in 24-well plate, 1 × 10^6^ cells per well in 6-well plate, and 4 × 10^6^ cells per 10-cm plate). For plasmid transfection experiments, a calcium phosphate protocol (*54*) was used; neurons were transfected on DIV12 and processed on DIV21 to DIV25. For circTulp4 overexpression experiments, the circTulp4 sequence was cloned into the Laccase2 MCS exon vector (*55*). In some cases, synaptic activity was induced in DIV21 neurons by adding bicuculline methiodide (50 μM; Sigma-Aldrich) and 4-aminopyridine (2.5 mM; Sigma-Aldrich) in the culture medium, as described by Hardingham *et al.* (*56*), for 3 hours.

Astroglial cultures enriched in astrocytes were prepared from CD1 mouse pups (P2) and maintained in Dulbecco’s modified Eagle’s medium supplemented with 10% heat-inactivated fetal bovine serum, penicillin (100 U/ml), streptomycin (100 μg/ml), and 2 mM l-glutamine (all from Gibco) at 37°C and 5% CO_2_. The astroglial precursors obtained from one pup were plated on a laminin-coated 10-cm plate. The medium was renewed 24 hours after plating and every 2 days after that. Upon confluency, cells were harvested.

### Cell lines and transient transfections

The mouse neuroblastoma-derived Neuro-2A cell line was grown in Dulbecco’s modified Eagle’s medium supplemented with 10% heat-inactivated fetal bovine serum, penicillin (100 U/ml), streptomycin (100 μg/ml), and 2 mM l-glutamine (all from Gibco) at 37°C and 5% CO_2_. Cells were regularly checked and tested negative for mycoplasma contamination. Neuro-2A cells were transiently transfected using Lipofectamine 2000 (Invitrogen), according to the manufacturer’s manual.

### Fluorescent in situ hybridization

Fluorescent in situ hybridization was performed using the QuantiGene ViewRNA miRNA ISH Cell Assay (Affymetrix) with a custom probe designed by the manufacturer and directed against the backsplice junction of circTulp4. Briefly, wild-type and transfected primary neurons grown on glass coverslips were fixed in 4% paraformaldehyde (PFA) in phosphate-buffered saline (PBS) for 1 hour at room temperature (RT). Upon washing 3× with PBS, cells were equilibrated 2× with cross-linking buffer QM for 10 min at RT, and the target was cross-linked with 0.16 M 1-ethyl-3-(3-dimethylaminopropyl)carbodiimide for 1 hour at RT and rocking. After washing 3× with PBS, cells were permeabilized with detergent solution QC for 10 min at RT and digested with working protease solution for 10 min at RT. A 2× and 3× washing steps with PBS were performed after permeabilization and after digestion, respectively. Cells were then hybridized with working probe solutions for 3 hours at 40°C in a dry incubator, washed 3× in wash buffer (soaking 3 min in between), and stored in storage buffer until the next day at 4°C. Cells were then washed 2× with wash buffer and consecutively hybridized with working preamplifier mix solution, working amplifier mix solution, and label probe working solution, each time for 1 hour at 40°C. After each hybridization step, 3× washing steps with wash buffer (soaking 3 min in between) were followed. For Fast Red development, cells were incubated with AP Enhancer for 5 min at RT, AP Enhancer was removed, and Fast Red substrate was incubated at 40°C for 45 min. Afterward, cells were washed 2× with PBS, fixed in 4% PFA in PBS for 10 min at RT, and rewashed 3× with PBS. Last, coverslips were stained with 4′,6-diamidino-2-phenylindole (DAPI) and mounted with an antifading VECTASHIELD medium (Vector Laboratories). Images were taken using a confocal microscope (Zeiss LSM 710).

### Subcellular fractionation

Mouse forebrains, or in some cases DIV21 primary neuronal cultures, were homogenized with a Potter-Elvehjem Teflon-glass homogenizer in ice-cold homogenization buffer [0.32 M sucrose, 2 mM EGTA, and 4 mM Hepes (pH 7.4), with protease and phosphatase inhibitor mixture]. All the following steps were performed at 4°C. The homogenate was first centrifuged at 1200*g* for 15 min, and the supernatant was centrifuged at 12,000*g* for 20 min. The resulting pellet (P2; crude synaptosomal sample) was washed once, resuspended in homogenization buffer, layered onto a discontinuous sucrose gradient containing 0.8 M/1.2 M sucrose, and centrifuged at 270,000*g* for 45 min. The band at the 0.8 M/1.2 M sucrose interface was collected, washed, and diluted with 0.32 M sucrose buffer and centrifuged at 20,000*g* for 20 min. The pellet containing the pure synaptosomes was stored at −80°C.

In some cases, pure synaptosomes were further processed to obtain a total SPM and a postsynaptic enriched (PSD-2T) fraction. In those cases, pure synaptosomes were subjected to a hypo-osmotic shock by adding 10 vol of 4 mM Hepes buffer (pH 7.4). Samples were gently shaken at 4°C for 30 min and centrifuged at 25,000*g* for 20 min, and the pellet was then resuspended to generate the total SPM fraction. To obtain the postsynaptic enriched fraction, SPM was solubilized with 0.5% Triton X-100 solution [containing 2 mM EGTA and 50 mM Hepes (pH 8.0), with protease and phosphatase inhibitor mixture], gently shaken at 4°C for 15 min, and centrifuged at 32,000*g* for 20 min to yield PSD-1T pellet. The PSD-1T pellet was again gently shaken at 4°C for 15 min and centrifuged at 32,000*g* for 20 min to yield the PSD-2 pellet. The PSD-2 pellet was resuspended in 0.2% SDS, 50 mM Hepes, and 2 mM EDTA to generate the PSD-2T fraction. Samples were stored at −80°C.

### In utero electroporation of mouse embryos

In utero electroporation of CD1 mouse embryos was performed as previously described (*57*). One to 2 μl of high-concentration plasmid DNA was in utero injected into the lateral ventricle of E15.5 mouse embryos using a glass micropipette and plunger (PCR micropipettes, 1 to 10 μl; Drummond). Electroporations were performed using an Electro Square Porator ECM830 and tweezertrodes (BTX Genetronics) to target the developing hippocampus as described (*58*), with five pulses (40 V, 50-ms duration, and 950-ms intervals) delivered to each embryo. Electroporated mice were euthanized 6 weeks after birth, and their brains were removed and subjected to electrophysiological or morphological analysis.

### Immunoblotting

Tissues or subcellular fractions were lysed in radioimmunoprecipitation assay buffer containing protease inhibitors and phosphatase when necessary and centrifuged, and protein concentration was quantified via Bradford assay (Bio-Rad). Samples were boiled in reducing sample buffer, separated by tris-glycine SDS-PAGE using TGX Stain-Free FastCast Acrylamide gels (Bio-Rad), and transferred to polyvinylidene difluoride membranes (Bio-Rad). After blocking in 5% milk (Roth) or bovine serum albumin (Sigma-Aldrich), membranes were incubated with primary and secondary (horseradish peroxidase–conjugated) antibodies. Chemiluminescence signals were acquired on a ChemiDoc imaging system and analyzed using Image Lab software (Bio-Rad). In most cases, Stain-Free imaging (Bio-Rad) was used as a loading control of total protein in each lane (*59*); in others, actin or glyceraldehyde-3-phosphate dehydrogenase (Gapdh) as housekeeping proteins was used for normalization. Antibodies are listed in table S2.

### RNA isolation and reverse transcription quantitative PCR

Tissues were homogenized in ice-cold TRIzol with an Ultra-Turrax disperser (IKA), whereas cultured cells were collected directly into ice-cold TRIzol for RNA preparation. Total RNA was obtained by combining chloroform extraction and purification with silica columns (RNeasy Mini kit, QIAGEN) according to the manufacturer’s instructions. After deoxyribonuclease I treatment, 350 ng (from cells) or 1 μg (from tissues) of total RNA was converted to cDNA using SuperScript III Reverse Transcriptase (Invitrogen) with random hexamer primers, according to the manufacturer’s protocol. Quantitative PCR (qPCR) reactions were performed on a 96-well format Bio-Rad PCR machine using FastStart Essential DNA Green Master Mix (Roche) in technical triplicates. To improve reproducibility and robustness, a combination of two reference housekeeping genes (*60*), ribosomal protein L19 (RPL19) and TATA-binding protein, were used to normalize RNA expression levels. Primer sequences used in qPCR reactions are listed in table S1.

### Immunofluorescence staining

Immunofluorescence stainings were performed as previously described (*61*). Briefly, neuronal and astroglial cultures were fixed with prewarmed 4% PFA containing 5% sucrose for 20 min at room temperature and then washed with PBS. Vibratome brain slices from transcardial-perfused mice were also used in some cases. Samples were permeabilized and blocked in 5% bovine serum albumin (Sigma-Aldrich) and incubated with primary antibodies (overnight at 4°C), followed by Alexa dye–conjugated secondary antibodies (Invitrogen). Samples were mounted in VECTASHIELD medium (Vector Laboratories). Antibodies are listed in table S2.

### Electrophysiological measurements

#### Slice preparation

Six-week-old mice were anesthetized and decapitated. Brains were removed into a chilled solution containing 110 mM choline-Cl^−^, 2.5 mM KCl, 2.0 mM NaH_2_PO_4_, 25 mM NaHCO_3_, 0.5 mM CaCl_2_, 7 mM MgCl_2_, 20 mM dextrose, 1.3 mM Na^+^-ascorbate, 3.1 mM Na^+^-pyruvate, and 4 mM kynurenic acid. The right hippocampus was dissected, and slices (350 μm in thickness) were cut transversally to the longitudinal axis in a vibratome and transferred to a chamber containing artificial cerebrospinal fluid: 125 mM NaCl, 2.5 mM KCl, 2.3 mM NaH_2_PO_4_, 25 mM NaHCO_3_, 2 mM CaCl_2_, 1.3 mM MgCl_2_, 1.3 mM Na^+^-ascorbate, 3.1 mM Na^+^-pyruvate, and 10 mM dextrose (315 mOsm). Slices were bubbled with 95% O_2_/5% CO_2_ and maintained at 30°C for >1 hour before experiments started. All salts were from Sigma-Aldrich.

#### Electrophysiological single-cell recordings

Recorded neurons were visually identified by fluorescence and infrared differential interference contrast (DIC) video microscopy. Whole-cell recordings were performed using microelectrodes (4 to 8 megohms) filled with 130 mM CsOH, 130 mM d-gluconic acid, 2 mM MgCl_2_, 0.2 mM EGTA, 5 mM NaCl, 10 mM Hepes, 4 mM adenosine 5′-triphosphate–tris, 0.3 mM guanosine 5′-triphosphate–tris, and 10 mM phosphocreatine. For evoked monosynaptic EPSC and IPSC recordings, a stimulating electrode was placed at the stratum radiatum to activate Schaffer collateral axons. EPSCs were isolated by voltage clamping pyramidal neurons at the reversal potential of the IPSC measured for each individual neuron (∼60 mV). In turn, IPSCs were recorded at the reversal potential of the EPSC (∼0 mV). Direct monosynaptic IPSC elicited in the presence of kynurenic acid was subtracted from the total IPSC. For recordings of the mEPSCs and mIPSCs, tetrodotoxin containing artificial cerebrospinal fluid (0.5 μM; Alomone Labs) was bath perfused. mEPSCs and mIPSCs were recorded at the reversal potential of inhibition (−60 mV) and excitation (0 mV), respectively, in gap-free mode. In experiments where the intrinsic responses to current pulses were evaluated, a potassium gluconate internal solution was used: 120 mM potassium gluconate, 4 mM MgCl_2_, 10 mM Hepes buffer, 0.1 mM EGTA, 5 mM NaCl, 20 mM KCl, 4 mM adenosine 5′-triphosphate–tris, 0.3 mM guanosine 5′-triphosphate–tris, and 10 mM phosphocreatine (pH 7.3; 290 mOsm). For recordings of AMPA and NMDA currents, picrotoxin (100 μM) was added to the bath solution. AMPA currents were recorded at −60 mV, while NMDA currents were obtained holding the voltage membrane at +40 mV and considered after a short delay from stimulation (60 to 70 ms) to isolate them from AMPA currents.

Recordings were obtained using Multiclamp 700B amplifiers (Molecular Devices, Sunnyvale, CA), digitized, and acquired at 20 kHz onto a personal computer using the pClamp10 software. Membrane capacitance and input resistance were obtained from current traces evoked by a hyperpolarizing step of 10 mV. Series resistance was typically 10 to 20 megohms, and experiments were discarded if higher than 40 megohms.

#### Extracellular in vitro recordings

To calibrate the input strength for the circuit properties experiments, field recordings were performed using patch pipettes (5 megohms) filled with 3 M NaCl placed on the stratum radiatum to record the field excitatory postsynaptic potentials (fEPSPs) in response to the Schaffer collateral stimulation.

### Behavioral characterization

Behavioral experiments were performed with three independent batches of male mice (age, 11 to 18 weeks), which were habituated to test room conditions 1 week before testing. All behavioral tests were performed during the light phase except for dark-light box tests. To minimize possible carryover effects of the different behavioral tests, the sequence of tests was arranged from least to most stressful in each behavioral pipeline (fig. S7A).

#### OF tests

In OF with high-illumination conditions, the analysis was carried out as described before (*62*). Briefly, mice were placed in a square OF arena (*WLH*, 45.5 cm by 45.5 cm by 39.5 cm), illuminated with 200 lux in the center, and surrounded by infrared beams (ActiMot, TSE GmbH).

In OF with low illumination conditions, mice were placed in a square OF arena (*WLH*, 50 cm by 50 cm by 50 cm), illuminated with 13 to 15 lux in the center. Behavior was recorded using webcams (Logitech BRIO), and resulting recordings were analyzed with ezTrack software (*63*). Behavior was recorded for 20 min, during which several parameters were analyzed, including distance traveled and time in the center (in percentage).

#### Modified SHIRPA screening

The primary behavioral observation screen is a modification of the procedure described by Irwin (*64*), in which the general health status and evaluation of basic neurological and motor functions are assessed. First, the mouse is placed in a clear cylinder to observe body posture and tremors. Then, it is transferred into an arena (*WLH*, 26 cm by 42 cm by 18 cm) where a Perspex sheet on the floor is marked with 15 squares. Observational profilings are obtained with a standardized semiquantitative score for each parameter. In particular, locomotor activity is recorded during the first 30 s by counting the number of squares being crossed.

#### Acoustic startle response and prepulse inhibition

The acoustic startle response (ASR)/prepulse inhibition (PPI) aims to characterize two dissociable functions: sensorimotor recruitment (ASR) and sensorimotor gating (PPI) (*65*, *66*). The latter reflects an animal’s ability to integrate and inhibit irrelevant sensory information successfully. The experimental apparatus consisted of an outer sound-attenuated chamber and an inner load cell platform that recorded the startle response (SR-LAB, San Diego Instruments). After an acclimation period of 5 min, the response to a 110-dB pulse was measured. For PPI assessment, the capacity of a weaker acoustic prepulse of varying intensities (67, 69, 73, or 81 dB) to attenuate a 110-dB pulse was calculated as a percentage score. For each acoustic prepulse trial, % PPI = 100 × (*S* − PPi_*S*) / *S*, where *S* is the basal startle amplitude at 110 dB and PPi is the startle amplitude after the prepulse presentation. The protocol consisted of 10 blocks, each including every trial condition in a pseudo-randomized order. ASR was also elicited 10 times at 110 dB without a prepulse to determine the baseline response and assess habituation effects.

#### Y-maze test

Spatial memory was investigated in the Y-maze, consisting of three identical arms (*WLH*, 5 cm by 30 cm by 15 cm) placed at 120° from each other and illuminated in the center with 100 lux, as described by Wall *et al.* (*67*). When tested in the Y-maze, normal mice prefer to explore the least recently visited arm, thus tending to alternate visits between the three arms. To do so, the mouse must maintain a record of the most recently visited arm and continuously update it. Therefore, alternation behavior is a measure of spatial working memory. During testing, each mouse was placed at the end of one arm and allowed to move freely through the maze during a 5-min session. Total arm entries were collected cumulatively over 5 min, accounting for locomotor activity. The percentage of spontaneous alternations—consecutive entries into all three arms without repetitions in overlapping triplet sets—was calculated as the ratio of total alternations to the total number of triplets × 100. The percentage of alternate arm returns—triplet sets that include a return into the first arm—was calculated as the ratio of alternate arm returns to the total arm entries × 100.

#### Novel object recognition test

An object recognition procedure with one 3-min sample trial and a 3-min test trial 3 hours later was performed as described by Genoux *et al.* (*68*) with minor modifications. The experimental arena settings are identical to those described for the OF under low illumination conditions. Significantly longer investigation durations of the novel object compared with the familiar one were taken as evidence for intact recognition memory. The recognition index was calculated as follows: index = investigation time novel / (investigation time familiar + investigation time novel).

#### Social discrimination test

Social discrimination was analyzed as previously described (*69*). The procedure consisted of two trials of 4 min exposures to an unfamiliar animal (ovariectomized 129Sv females). Before the first exposure, the test animal was allowed to move freely in a new cage for a 2-hour period, after which an unfamiliar animal was presented. After a retention interval of 2 hours, the test animal was reexposed to the now familiar animal, together with an additional new, unfamiliar animal. The duration of the investigatory behavior of the test animal toward the unfamiliar animal was recorded by a trained observer with a handheld computer. A social recognition index was calculated as the ratio between the time spent investigating the unfamiliar mouse to the time spent investigating both the familiar and unfamiliar mice.

#### Grip strength

The grip strength–meter system determines the grip strength of the limbs, i.e., muscle strength of a mouse. The device exploits the tendency of a mouse to grasp a horizontal metal grid while being pulled by its tail. During the trial setup, the mouse grasps a particular adjustable grid mounted on a force sensor. The mouse is allowed to catch the grid with either two or four paws. Three trials were undertaken for each mouse and measurement within 1 min. The mean values are used to represent the grip strength of a mouse. All experimental equipment was thoroughly cleaned with Pursept-A and dried before subsequent tests.

#### Accelerating rotarod

The rotarod (Bioseb, Chaville, France) was used to measure motor coordination, balance, and motor learning ability (*70*). The machine is placed in an environment with minimal stimuli, such as noise and movement. The rotarod device is equipped with a computer-controlled motor–driven rotating rod and consists of a rotating spindle and five individual lanes for each mouse. Magnetic sensors are used to detect when a mouse falls from the rotarod. Mice are placed perpendicular to the axis of rotation, with the head facing the direction of the rotation, at an accelerating speed from 4 to 40 rpm for 300 s with 15 min between each trial. In the motor coordination testing, mice were given three trials at the accelerating speed for 1 day. The mean latency to fall off the rotarod during the trials was recorded and used in subsequent analysis. In addition, the reason for the trial end (falling, jumping, or rotating passively) is recorded. Before the start of the first trial, mice were weighed.

#### Auditory brainstem response

Auditory brainstem response (ABR) is an auditory evoked potential and one of the critical methods in mice’s noninvasive assessment of hearing sensitivity. Mice anesthetized with ketamine/xylazine are transferred onto a heating blanket in the acoustic chamber, and three subcutaneous needle electrodes are placed (*71*). Since the stimuli are present as free-field sounds from a loudspeaker, the heads of the mice are placed at a calibrated distance. This distance is determined by calibrating the microphone daily with white noise before the measurements begin. For threshold determination, the clicks (0.01-ms duration) or tone pips (6, 12, 18, 24, and 30 kHz of 5-ms duration and 1-ms rise/fall time) stimuli over a range of intensity levels from 5- to 85-dB sound pressure level (SPL) in 5-dB steps produced by Tucker Davis Technologies hardware with customized software, provided by Welcome Trust Sanger Institute, are used. The sound intensity threshold is chosen manually from the first appearance of the characteristic waveform. Once each mouse is placed in the acoustic chamber for recording, the following procedure is used: (i) initial ABR test: A response to 70 dB click broadband stimuli is recorded to ensure correct setup; (ii) determination of ABR hearing threshold: A series of click-evoked ABRs is recorded in response to broadband click stimuli ranging from 0- to 85-dB SPL in 5-dB intervals; (iii) determination of tone-evoked ABR thresholds: Tone-evoked ABRs are recorded for a set of frequencies (6, 12, 18, 24, and 30 kHz) over sound levels ranging from 0- to 85-dB SPL in 5-dB intervals; and (iv) the recording of the response to 70-dB click broadband stimuli is repeated to ensure consistency of measurement.

#### Hot plate

The mice were placed on a metal surface maintained at 52° ± 0.2°C (hot plate system, TSE GmbH, Germany). Locomotion of the mouse on the hot plate was constrained by a 20-cm-high Plexiglas wall to a circular area with a diameter of 28 cm. Mice remained on the plate until they performed one of three behaviors indicative of nociception: hind paw lick (h.p. licking), hind paw shake/flutter (h.p. shaking), or jumping. We evaluated only the hind paw but not the front paw responses because fore paw licking and lifting are components of normal grooming behavior. Each mouse was tested only once since repeated testing leads to profound changes in response latencies. The latency was recorded to the nearest 0.1 s. A cutoff time of 30 s was used to avoid tissue injury.

#### Beam ladder test

In the beam ladder, mice were required to traverse a narrow horizontal beam ladder of metal beams of 1 mm in diameter with different and irregular distances to each other. Traversing time and front and hind paw slips were recorded manually, and the means of three trials of each mouse were used for data analysis.

#### Beam balance test

In the beam balance test (also known as the raised-beam test or beam walk), mice were trained to traverse a distance of 90 cm on a series of elevated, narrow beams (diameters of beams 1 to 4: square, 20 mm; round, 22 mm; square, 12 mm; round, 15 mm) to reach their respective home cage. Traversing time, foot slips, and falls were recorded manually, and the means of three trials of each mouse were used for data analysis.

#### Marble burying

Mice were placed into a housing cage filled with corn cob bedding (5 cm in height) containing 18 glass marbles evenly distributed over the surface of the corn cob layer, as described by Deacon R (*72*). After the cages had been covered with cage lids, animals were allowed to roam the cages freely for 30 min. At the end of the test period, the number of buried marbles was assessed by an observer blind to the genotype.

#### Elevated plus maze

Anxiety-related behavior was measured by means of the elevated plus maze. The apparatus was made of gray polyvinyl chloride and consisted of a plus-shaped platform with four intersecting arms, elevated 37 cm above the floor. Two opposing open (30 cm by 5 cm) and closed arms (*WLH*, 30 cm by 5 cm by 15 cm) were connected by a central zone (5 cm by 5 cm). Animals were placed in the center of the apparatus facing the closed arm and allowed to explore the maze for 5 min freely. Parameters of interest included open-arm time and open-arm entries.

#### Forced swim test

The forced swim test represents a well-established paradigm to assess helplessness, a component of depressive-like behavior in mice (*73*). Animals were carefully placed into a glass beaker (diameter, 14 cm; height, 30 cm) filled with tap water (23° ± 1°C) to a height of 18 cm so that the mouse could not touch the bottom with its hind paws or tail. The duration of the test was 6 min and was recorded. The time spent immobile and the time spent struggling were scored by an experienced observer, blind to the genotype of the animals. The freely available Solomon Coder software (ELTE TTK) was used for scoring. Only the last 4 min of each trial were considered for the analysis because most mice were very active at the beginning of the forced swim test, which is a potential confounding factor.

#### Acute restraint stress

Acute restraint stress was performed as in Zimprich *et al.* (*74*) with minor modifications. Briefly, mice were restrained in well-ventilated 50-ml tubes for 30 min during the dark phase. After the restraint period, the mouse was transferred into a clean animal housing cage.

#### Dark-light box test

The dark-light box test was used to assess anxiety-related behavior as described by Mozhui *et al.* (*75*), with minor modifications. The test was performed during the dark phase in a rectangular apparatus consisting of an aversive brightly lit compartment (*WLH*, 33.5 cm by 50 cm by 55 cm; two of three of the total surface; 800 to 1000 lux) and a more protective dark compartment (*WLH*, 16.5 cm by 50 cm by 55 cm; one of three of the total surface; 15 lux). At the start of the test, all mice were placed in the dark compartment and were allowed to freely explore the apparatus for 5 min. The sessions were recorded, and the Solomon Coder software (ELTE TTK) was used to calculate latency, time in the lit compartment, and the number of transitions. Zone entries were counted if at least the two front paws and half of the animal’s body were inside the compartment.

### Analysis of neuronal morphology

CD mice were crossed with the Thy1EGFP line (*50*) to genetically mark and evidence the morphology of a sparse population of neurons through EGFP protein expression.

#### Brain slice preparation

Mice (10 weeks old) carrying the Thy1EGFP transgene were anesthetized with isoflurane (Piramal Critical Care) and perfused transcardially with a peristaltic pump for 1 min with PBS to drain out the blood, followed by 5 min with fixation solution [4% PFA in PBS (pH 7.4)]. Brains were removed and left for 24 hours in fixation solution at 4°C. Subsequently, brains were washed with PBS and embedded in a warm solution of 4% (w/v) agarose (Invitrogen) in PBS to perform cross sections of 150 μm in thickness in the hippocampal area with a vibratome (VT1000 S, Leica). Brain sections were stored at −20°C in cryopreservation solution [20% (v/v) glycerol and 30% (v/v) ethylene glycol in PBS].

#### Image acquisition and analysis

Images of brain slices were taken using a Zeiss LSM 710 confocal microscope (Plan-Apochromat 20×/0.8 M27 and Plan-Apochromat 40×/1.4 oil DIC M27 oil immersion objective for dendritic arborization and spines, respectively) and processed in Neurolucida 360 software (MBF Bioscience). This software uses a *z*-stack of confocal images to generate three-dimensional digital representations of neurons, resulting in accurate reconstructions of the dendritic tree and the dendritic spines. As a first step, the pyramidal neurons were traced on the brain slices to select neurons with no evident missing fragments and adequate fluorescence levels.

For the dendritic arborization analysis, the images were acquired using *z*-steps of 0.5 μm. In all cases, the number of *z*-steps used included the structure of neurons spanned on the *z* axis. Subsequently, these images were imported into Neurolucida 360 to reconstruct the neuronal soma and the dendritic arbor in three-dimensional in a semiautomatic manner. Soma volume, complexity, and total dendritic length were the parameters to be studied. The dendritic tree complexity data were estimated by the Sholl analysis, in which the number of intersections between the dendrites and a series of concentric circles starting from the soma is measured.

For dendritic spines, the images were acquired using *z*-steps of 0.1 μm and passed through a deconvolution process performed with ImageJ software using the Iterative Deconvolve 3D plugin. The three-dimensional reconstruction of dendritic spines was carried out using an automatic tool of the software, which detects protrusions according to reported parameters corresponding to hippocampal CA1 pyramidal neurons (*76*). Quantification of the density and morphological aspects of dendritic spines were extracted using Neurolucida 360 Explorer (MBF Bioscience). This tool returns the length of dendrites, the number of spines, their volume, and their morphological classification according to automatically set parameters corresponding to neuron type.

### Label-free protein quantification of pure synaptosomal fractions

#### Proteolytic digestion

Pure synaptosomal protein fractions corresponding to 20 μg of protein were dissolved in lysis buffer and processed according to a filter-aided sample preparation (FASP) protocol modified as described by Distler *et al.* (*77*). Unless stated otherwise, all steps were automated on a liquid-handling workstation equipped with a vacuum manifold (Freedom EVO 150, Tecan) using an adaptor device constructed in-house. Briefly, protein samples were lysed and reduced by shaking for 30 min at 37°C and then loaded on centrifugal filter units (30-kDa molecular weight cutoff; Millipore). After removal of the detergents by washing twice with wash buffer, proteins were alkylated with 50 mM iodoacetamide in 8 M urea/0.1 M tris (pH 8.5) (z20 min at RT), followed by two washes with wash buffer to remove excess reagent. Buffer was exchanged by washing three times with 50 mM ammonium bicarbonate (ABC) containing 10% acetonitrile. After three additional washes with 50 mM ABC/10% acetonitrile, performed by centrifugation to ensure quantitative removal of liquids, proteins were digested overnight at 37°C with 500 ng of trypsin in 40 ml of the same buffer. Tryptic peptides were recovered by centrifugation, followed by two additional extraction steps with 40 ml of 50 mM ABC and 40 ml of 1% trifluoroacetic acid, respectively. Aliquots of the combined flow-throughs were spiked with yeast enolase 1 tryptic digest standard (10 fmol/ml; Waters Corporation) for quantification purposes as described by Silva *et al.* (*78*) and directly subjected to analysis by liquid chromatography coupled to electrospray MS (LC-MS).

#### Lysis buffer

The lysis buffer composition was 7 M urea (Sigma-Aldrich), 2 M thiourea (Sigma-Aldrich), 10 mM dithiothreitol, 2% CHAPS, and 0.1 M tris (pH 8.5).

#### Wash buffer

The wash buffer composition was 8 M urea, 10 mM dithiothreitol, and 0.1 M tris (pH 8.5). 

#### LC-MS analysis

Nanoscale reversed-phase ultraperformance liquid chromatography separation of tryptic peptides was performed with a nanoAcquity ultraperformance liquid chromatography system equipped with a Symmetry C18 5-µm, 180-mm by 20-mm trap column and an HSS T3 C18 1.8-µm, 75-µm by 250-mm analytical column maintained at 45°C (Waters Corporation). Injected peptides were trapped for 4 min at a flow rate of 8 ml/min and 0.1% trifluoroacetic acid and then separated over 120 min at a flow rate of 300 nl/min with a gradient comprising two linear steps of 3 to 35% mobile phase B in 105 min and 35 to 60% mobile phase B in 15 min, respectively. Mobile phase A was water containing 0.1% formic acid, while mobile phase B was acetonitrile containing 0.1% formic acid. MS analysis of tryptic peptides was performed using a Synapt G_2_-S quadrupole time-of-flight MS equipped with ion mobility option (Waters Corporation). Positive ions in the mass/charge ratio (*m*/*z*) range of 50 to 2000 were acquired with a typical resolution of at least 20,000 full width at half maximum, and data were lock mass–corrected after acquisition. Analyses were performed in the ion mobility–enhanced data-independent acquisition mode with drift time–specific collision energies as described in detail by Distler *et al.* (*77*). Continuum LC-MS data were processed for signal detection, peak picking, and isotope and charge state deconvolution using Waters ProteinLynx Global Server (PLGS) version 3.0.2. A custom database was compiled for protein identification by adding the sequence information for yeast enolase 1 and porcine trypsin to the UniProtKB/Swiss-Prot mouse proteome and appending the reversed sequence of each entry to enable the determination of false discovery rate (FDR). Precursor and fragment ion mass tolerances were automatically determined by PLGS 3.0.2 and were typically below 5 parts per million (ppm) for precursor ions and below 10 ppm (root mean square) for fragment ions. Carbamidomethylation of cysteine was specified as fixed and oxidation of methionine as variable modification. One missed trypsin cleavage was allowed. Minimal ion matching requirements were two fragments per peptide, five fragments per protein, and one peptide per protein. The FDR for protein identification was set to 1% threshold.

#### Quantification

The freely available software ISOQuant (https://proteomics.uni-mainz.de/software/) was used for postidentification analysis including retention time alignment, exact mass, and retention time and ion mobility clustering, data normalization, isoform/homology filtering, and calculation of absolute in-sample amounts for each detected protein according to the Top3 quantification approach. Only peptides with a minimum length of seven amino acids identified with PLGS scores above or equal to 5.5 in at least two runs were considered. FDR for both peptides and proteins was set to 1% threshold, and only proteins reported by at least two peptides were quantified using the Top3 method. The parts per million abundance values [i.e., the relative amount (w/w) of each protein with respect to the sum over all detected proteins] were log_2_-transformed and normalized by subtraction of the median derived from all data points for the given protein. Significant changes in protein abundance were detected by moderated *t*-statistics with an empirical Bayes approach and FDR-based correction for multiple comparisons (*79*). For this purpose, the Bioconductor packages “limma” (*80*) and “q-value” (*81*) were used in RStudio, an integrated development environment for the open-source programming language R. The MS proteomics data have been deposited to the ProteomeXchange Consortium via the PRIDE (*82*) partner repository with the dataset identifier PXD045787.

### In silico prediction analysis of miRNA binding sites

The potential binding of miRNAs to circTulp4 and other potential target transcripts was predicted with miRDB (*34*), a database for miRNA target prediction and functional annotations. miRDB is available online at https://mirdb.org/. All the predicted targets have target prediction scores between 50 and 100. The higher the score, the more confident the prediction. We considered only predicted targets with prediction scores of >70.

### RNA sequencing

The cortex, hippocampus, and cerebellum were dissected from wild-type and CD mice. RNA was extracted using the RNeasy mini kit with in-column deoxyribonuclease treatment (QIAGEN) according to the manufacturer’s instructions. Thirty nanograms of RNA was used as input for preparing 3′ RNA-seq libraries following CelSeq2 protocol (*83*), changing the unique molecular identifier to six bases. Sequencing was performed on Illumina NextSeq 500 system.

Raw reads were aligned to the *Mus musculus* genome (version mm10) using STAR (*84*). Reads were quantified using End Sequence Analysis Toolkit (*85*) for 3′ RNA libraries. Differential gene expression analysis was done with edgeR (*86*). We excluded from the analysis genes that do not reach 5 cpm in at least two samples. Reads were normalized by the trimmed mean method for each experimental group before differential expression analysis. The RNA-seq data have been deposited in Gene Expression Omnibus (GEO) with the dataset identifier GSE246304.

### LC-MS/MS analysis and peptide identification

Parallel reaction monitoring (PRM) analyses were carried out with a Q-Exactive Plus hybrid MS (Thermo Fisher Scientific, San Jose, CA, USA) coupled to an Ultimate 3000 UHPLC system (Dionex) with 0.1% formic acid as mobile phase A and 95% acetonitrile/0.1% formic acid as mobile phase B. The theoretical synthetic peptides (NLRGHNSESCK, GHNSESCK, and SPSRTFQS, 1 pmol each) were analyzed to collect MS/MS reference spectra. A mouse brain protein digest (1 μg) was injected onto a nanocolumn (15 cm in length and 75 μm in diameter; PicoTip Emitter, New Objective, USA) self-packed with C18 (1.9-μm particles, Dr. Maisch GmbH, Germany) and then subjected to a 65-min gradient elution at a flow rate of 300 nl/min, 2% mobile phase B (0 to 5 min), elution with 2 to 40% mobile phase B (5 to 40 min), 40 to 80% mobile phase B (40 to 50 min), and 2% mobile phase B (50 to 65 min). The MS was set to collect in PRM mode with an inclusion list for the theoretical peptides (415.53153, 431.17961, and 455.22487) following a full MS scan (350 to 1500 *m*/*z*). Full scans were conducted at a resolution of 70,000 at 200 *m*/*z* with an automatic gain control (AGC) setting of 3 × 10^6^ and a maximum injection time of 200 ms. PRM scans were collected at a resolution of 17,500 at 200 *m*/*z* with AGC setting of 2 × 10^5^ and a maximum IT of 100 ms. The isolation window was set to 4.0 *m*/*z*, and normalized collision energy was 27. Raw files were searched with SEQUEST in Proteome Discoverer 2.2 against the Swiss-Prot mouse protein sequence database that included the three peptide sequences. Search parameters were 10-ppm mass tolerance for peptide precursors and 20-mDa (higher collisional dissociation) mass tolerance for fragment ions, carbamidomethylation of cysteine residues as fixed modification, and oxidation of methionine as variable modification. The decoy search results indicated a <1% FDR.
