# Supplementary material for: A brain-enriched circular RNA controls excitatory neurotransmission and restricts sensitivity to aversive stimuli
Source: Sci Adv. 2024 May 24;10(21):eadj8769. doi: 10.1126/sciadv.adj8769 (PMC11122670; doi:10.1126/sciadv.adj8769)
Supplement: Supplementary file 1 — Figs. S1 to S10 Tables S1 and S2 Legends for data S1 and S2 [file sciadv.adj8769_sm.pdf]

Science Advances
AAAS

Supplementary Materials for

A brain-enriched circular RNA controls excitatory neurotransmission and restricts sensitivity to aversive stimuli

Sebastian A. Giusti *et al.*

Corresponding author: Damian Refojo, drefojo@ibioba-mpsp-conicet.gov.ar

Sci. Adv. **10**, eadj8769 (2024)
DOI: 10.1126/sciadv.adj8769

The PDF file includes:

Figs. S1 to S10
Tables S1 and S2
Legends for data S1 and S2

Other Supplementary Material for this manuscript includes the following:

Data S1 and S2

Giusti, *et al.* Supplementary Figure 1

circTulp4 sequence (5’-3’)
Length: 1946 nt

AGTTGTAAGAGTCCATCCAGGACCTTCCAGTCATGAATAATCTGATGGCTCCTGAATTAACCGGGAAAACAAACATATCAAGTGCCATTTGAAG
ACTCTGTCTATCTATGTAAAACCTTTTCTGCACATAGAAGCTTTTCCATAAGAAGACATTCTGAATTTTGCAACTGATGAAGATTAAGCATCAGC
TGGAGCACCTTTCCACTGTTGGGGTTGGGAGTCTGTGGAGAACAGTCTGTCACTAATGTCAGATTTTCCTTACAGTGTTCATAAACAAAAGCC
AGTTTGCAAAAGAAAAAATTGCACAGATTAACCCTAAAGAATAGCTCCAGTGTAAAGCAGGGGCAGACCTTAAAACTCTGAACCGGAGCTC
AGTGACTTTTCCTGTGGTTGTAGCAGGAGTGAGGGGACTGATCTGAAAGGAACAGATTCCTTTGTGTCTTCAGCTATCGGAAGTTTTTTATTTA
TTGTTTATCTTTTTTCTTTTCTGCATATATATGCCATTTTAAAATACTAATTGGAACTGTCAGTTACAAAATAAATATCAAGAAAAGCCTTTTTTGG
TCCAAAGGTGTGAACAGGCTTGCAGGTGAACAGGAAGACATCTTTGGTAAAGCTTGGACCGGTCTTGGGAGATGGTGCATCTTGGAGGGCT
CTGCTACACGCCTAGGTGGCTGGTTTAAAGGCTTGCATCGCAGACGGAGACTAATTAATAGACACAGTTCTAAGATTGCTTTTTCATTAACATA
GAAACTAGAGAAAGGAGAAACAGAAGCCTGCAGCCTAGTCTGTGTAAGCAAGAAGTAGGACCAAACTGAGGAAACTACTTGGTCCATTTAG
ATAGCAGTTTATTCATACTCAGTGACCCGCAGGCCTCCACCTCTGCTTGAGGGAAGGGCTTTGCTCCAGTCTCTGTGGCACTGAGGGTGGTTC
CAGCCCATGGAGGAGTCATTCTAGGAAGCCCTGTGTTCCTAGGGACACAGGGCCAGGCTTTGAGACAGGAAGCTTCTGGCTGTGAGCAGTG
GGGGAAAGAGTGATTTTCTTGTTAAAGCTTTGACCATTGTCTGCATGAGCTCTGGTGTGACTTTGCACGTTAGTGTGCCTTTCCCCTTATGCAA
CCTTTTCCAGCTTACAGCAGAAACTTGCCGAGTTCAGAAAACGTGCCAGAGGGTGGCTTCAGAGGGAAGATGATCTTGTGTGATCAGTCTCT
GCACTTGAACTATTGAATAGAGAAATCCAGCTAGAGGAATTCTTACCGCCTTAAGTTACTTGAAATCTATGTGTTTGTAACCCTTTGTCTCTGGA
ATTACATTACAAAAAAAACTGGAATCTCAGGCTGAGAATAACGAGGCTGAGTAAAAGCGAAGAGAACTGCCTCTTCATCATCACTTACTAACA
GCTCTTTCTCCAAAGGATTGGTGTGGTTTCCCGCTAAGAACTTGAAAATGAGAACGGACCCTGTGTATTTTTAGGCATTACCTTTCTTCGCCGA
CTGACGTCTTTTATAGAGGAGTTTTTTCACTATGCATTTGGTGGAGCTTTATAAGCTATTGACCTAATTGGACTCTAGATCAGTTGTAACTAAAG
GAGAAAAAAACAAACCAACGGAACCCAACCACAAAAATAAGCCAATAAAAGAACTTGGTTTGAAATTCCTCAGTACTTTTAAAGTGAAATAC
TTCATTGAAAAAAGTATGTATGCAGCAGTGGAACATGGGCCTGTGCTTTGCAGCGATTCCAACATCCTCTGCCTGTCCTGGAAGGGGCGTGTT
CCCAAGAGTGAGAAGGAGAAACCTGTGTGCAGAAGGCGCTACTATGAAGAGGGATGGTTGGCCACAGGCAATGGGCGAGGTGTGGTGGGA
GTGACTTTCACCTCGAGTCACTGTCGCAGAGATAGGAGTACACCACAGAGAATAAACTTCAACCTGCGAGGCCACAACAGTGAG

Genomic coordinates (Genome assembly): Chromosome 17: 6,187,486 - 6,189,431 forward strand (GRCm39)

Previous names: mmu_circ_0000723 (circBase)

Fig. S1. CircTulp4 sequence. CircTulp4 is a 1946-nucleotide-long exonic circRNA. In circBase it is identified as mmu_circ_0000723.

Giusti, *et al.* Supplementary Figure 2

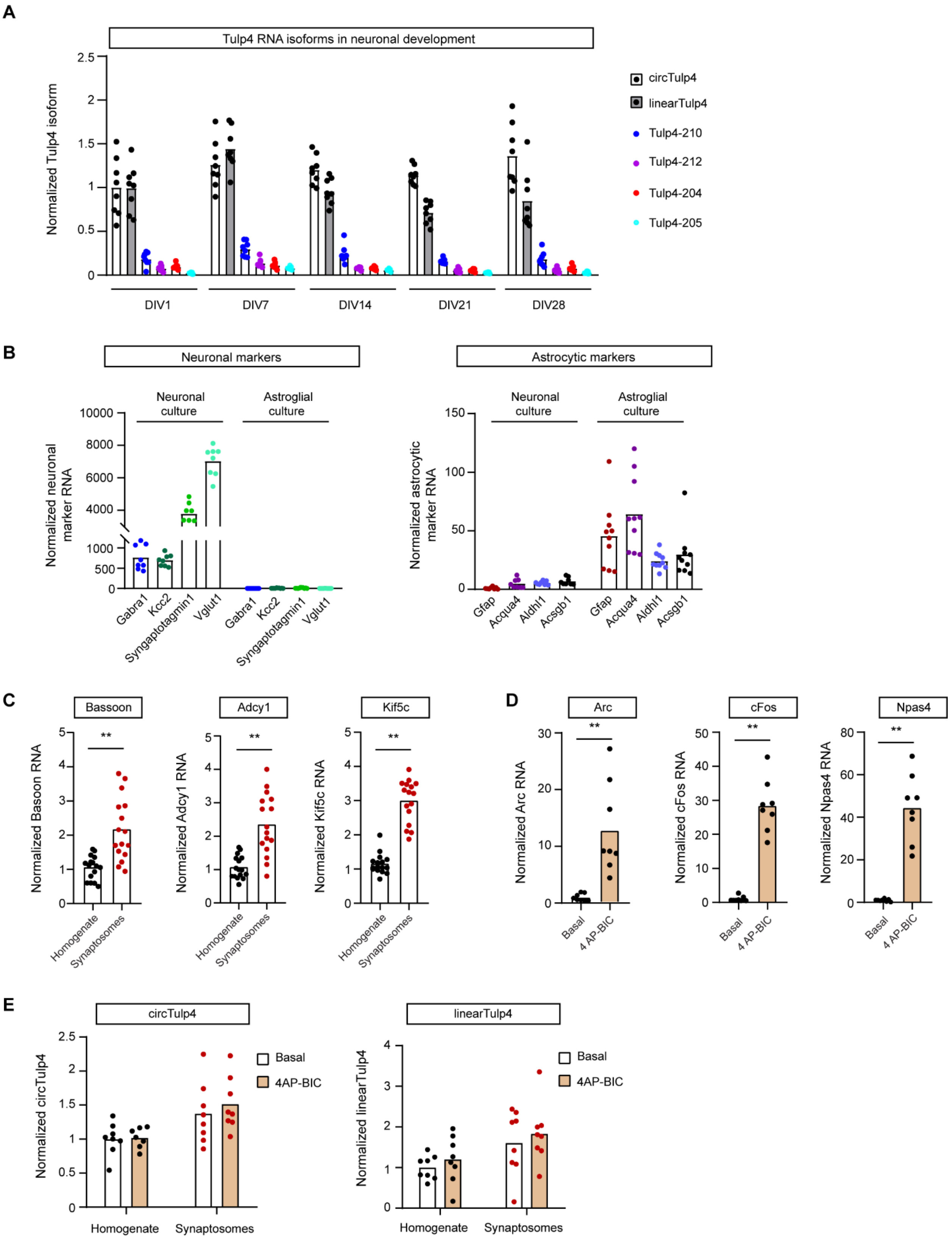

Fig. S2. Tulp4 RNA isoforms in neurons, astrocytes, and synaptic fractions. (**A**-**E**) Relative quantification by RT-qPCR. All values were normalized with the average of two housekeepers from the same sample: RPL19 and TATA-binding protein. (**A**) Tulp4 RNA isoforms in primary forebrain mouse neurons at different maturation time points (days in vitro, DIV). Linear Tulp4, the sum of Tulp4-201 and -209, represents the major linear isoform. The average value for normalized circTulp4 at DIV1 was arbitrarily set in '1' (n=8 independent cultures followed from DIV1 to DIV28). (**B**) Neuronal and astrocytic markers show that neurons (DIV21) and astroglial cultures have a high degree of enrichment in neurons and astrocytes, respectively (n=8 independent neuronal cultures, n=9 independent astroglial cultures). (**C**) Synaptic markers validate the preparation of synaptosome fractions from DIV21 primary neurons (n=16 homogenate-synaptosome independent pairs; paired t test). (**D**) Neuronal activity markers measured in homogenates show that the 4-aminopyridine / bicuculline (4AP-BIC) treatment for 3 h induces synaptic activity (n=16 homogenates from independent cultures; paired t test). (**E**) Synaptic stimulation of DIV21 primary neurons with 4AP-BIC treatment for 3 h did not affect circ- and linearTulp4 abundance or synaptic localization (n=15 homogenate-synaptosome independent pairs; two-way RM ANOVA and Sidak's multiple comparison test). In all cases, columns represent mean values. $^{**}P < 0.01$.

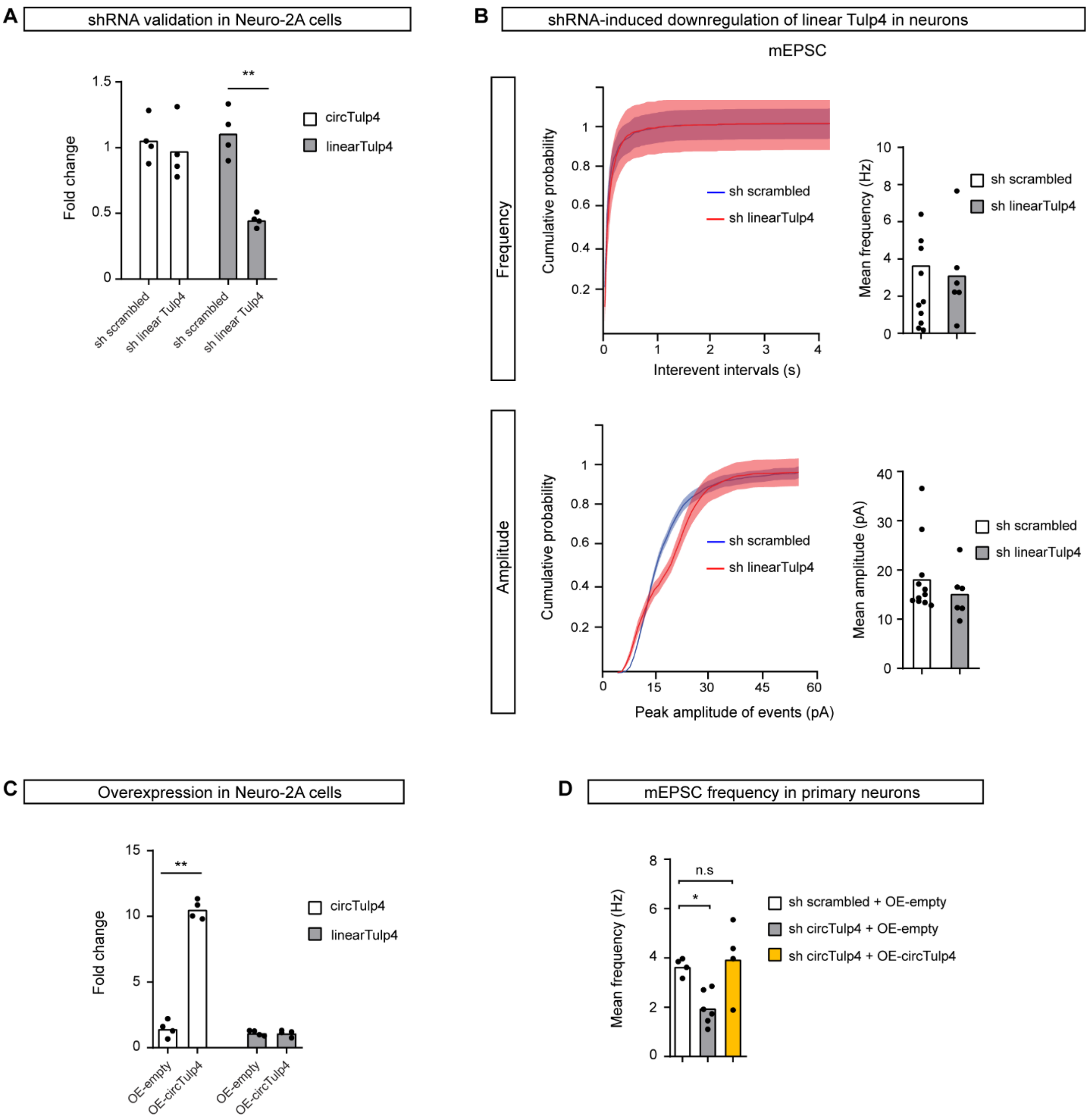

Fig. S3. Specificity controls for the effect of sh-circTulp4 on the frequency of mEPSCs. (A-B) Downregulation of linear Tulp4 does not affect miniature excitatory post-synaptic currents in primary neurons. (**A**) Loss-of-function experiments were performed with a shRNA vector (sh-linear Tulp4) that selectively downregulates linear Tulp4 (Tulp-201 and -209 transcripts) without affecting circTulp4. A scrambled shRNA sequence (sh-scrambled) was used in the control condition. The efficiency and specificity of the shRNAs were tested in Neuro-2A cells (Two-way RM ANOVA and Sidak's multiple comparison test, n= 4), **P < 0.01. (**B**) Mouse primary hippocampal neurons were cotransfected at DIV 12-13 with RFP and either sh-scrambled or sh-linear Tulp4. Electrophysiological recordings of transfected neurons were performed at DIV 21-

25. Linear Tulp4 downregulation did not affect the frequency or amplitude of miniature post-synaptic excitatory currents (mEPSCs) measured by whole-cell recordings (t test with Welch's correction, n= sh-scrambled, 11 cells; sh-linear Tulp4, 6 cells). Recorded cells were from two independent primary cultures. **(C-D)** Overexpression of circTulp4 rescues the diminishment of mEPSC frequency induced by sh-circTulp4. (**C**) Validation of the overexpressing (OE) construct in Neuro2A cells. As a control, the empty vector of the OE construct was used (Two-way RM ANOVA and Sidak's multiple comparison test, n= 4), **P < 0.01. (**D**) Mouse primary hippocampal neurons were cotransfected at DIV 12-13 with RFP and one of the following three combination of constructs: sh-scrambled and OE-empty, sh-circTulp4 and OE-empty, or circTulp4 and OE-circTulp4 (rescue condition). Electrophysiological recordings of transfected neurons were performed at DIV 21-25. The frequencies of miniature post-synaptic excitatory currents (mEPSCs) were measured by whole-cell recordings. The OE-circTulp4 construct restores the reduction in mEPSCs´ frequency induced by the sh-circTulp4 (ANOVA and Dunnett’s multiple comparison test, n= 4-6). *P < 0.05, n.s: not significant. In all cases, columns represent mean values.

Giusti, *et al.* Supplementary Figure 4

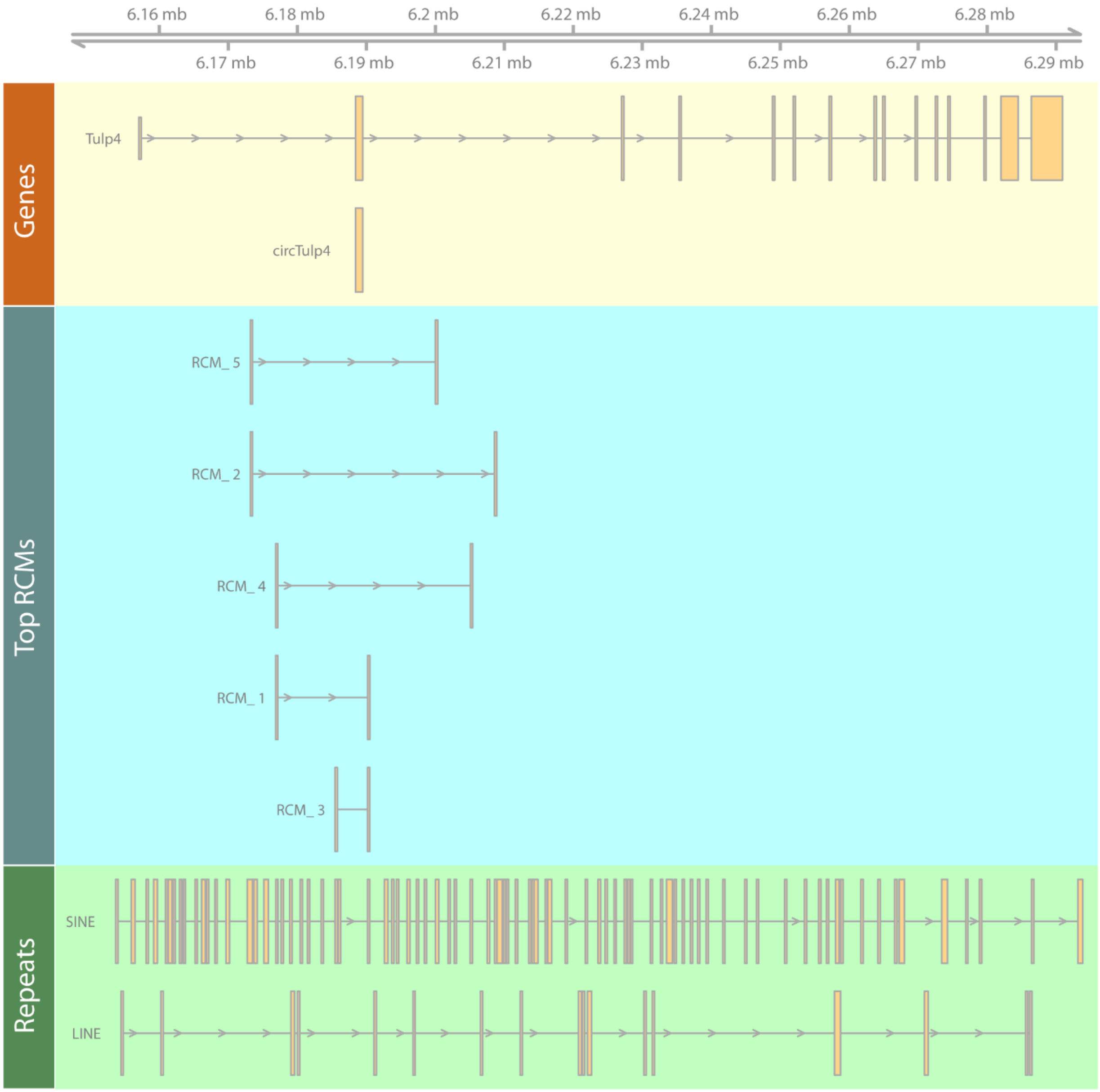

Fig. S4. Reverse Complementary Matches (RCMs) in the introns flanking circTulp4 sequence. Top RCMs, ranked based on the length and percentage of complementarity, are represented in the scheme. All RCMs found, with their location and sequence, are ranked in table S1. Most mapped RCMs overlapped with short interspersed nuclear elements (SINEs).

Giusti, *et al.* Supplementary Figure 5

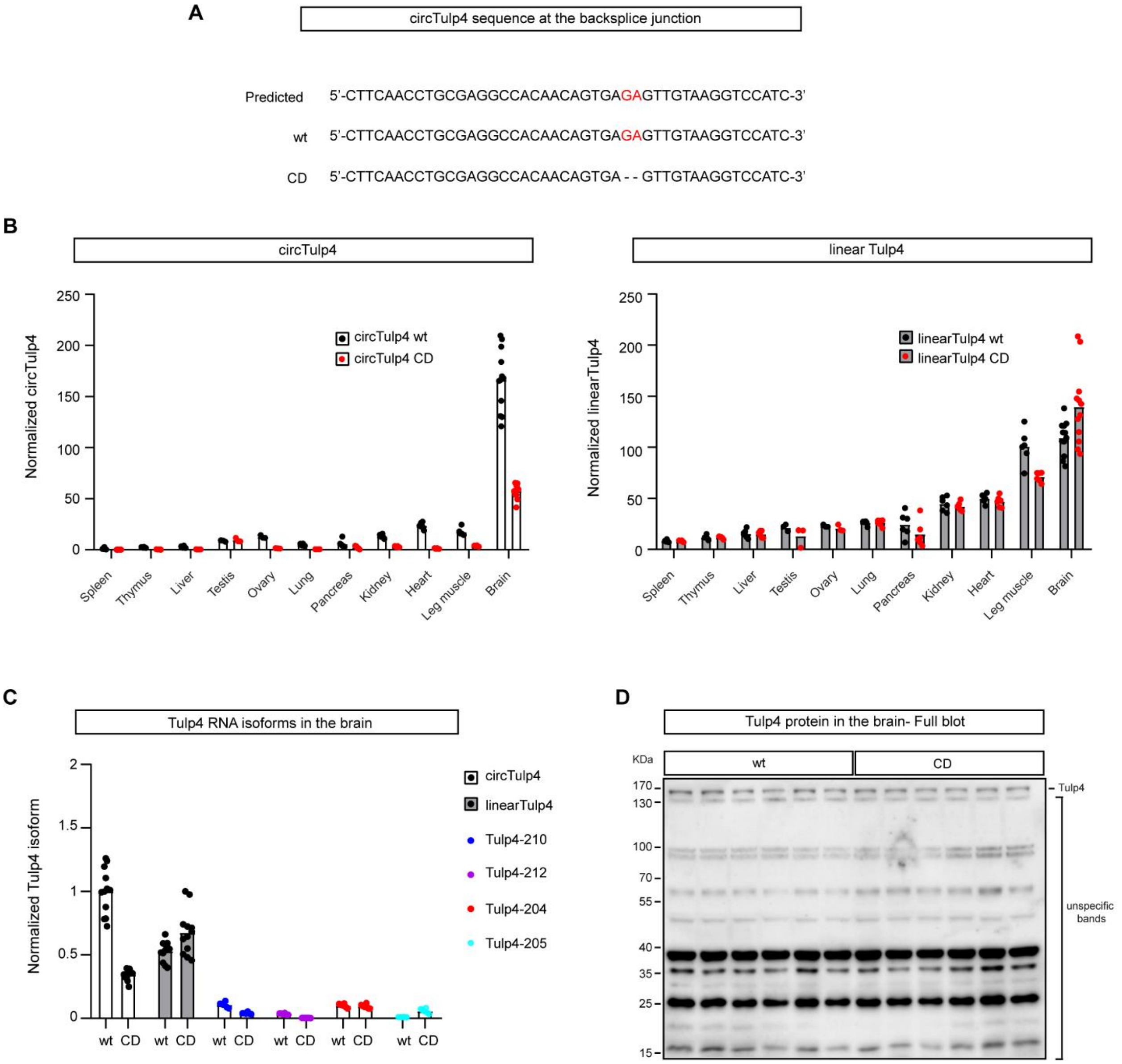

Fig. S5. Tulp4 RNA isoforms in circTulp4 deficient (CD) mice. (**A**) Sequences of circTulp4 RT-PCR products from wt and CD mice using circTulp4-specific primers and containing the backsplice junction were obtained by Sanger sequencing. Alignment of CD against wild type (wt) mouse data shows that upon mutagenesis of the splice acceptor site of circTulp4, shown in red letters, a new circular variant is generated using the following AG cryptic site in the sequence. (**B** and **C**) Relative quantification of Tulp4 RNA isoforms by RT-qPCR. All values were normalized with the average of two housekeepers from the same sample: RPL19 and TATA-binding protein. (**B**) circTulp4 (*left*) and linear Tulp4 (*right*) in different mouse tissues. The average value for normalized circTulp4 in the spleen was arbitrarily set in '1' (n= 12 for the brain, n=3 for testis and ovary, n=6 for other organs). (**C**) Tulp4 RNA isoforms in brain samples of wt and CD mice (n= 6 mice/genotype, except for circ- and linear Tulp4 with n=12 mice/genotype). (**D**) Full-blot from Western blot shown in Fig. 3B of brain samples with Tulp4 antibody. Predicted molecular weight of mouse Tulp4: 169.6 KDa.

Giusti, *et al.* Supplementary Figure 6

A

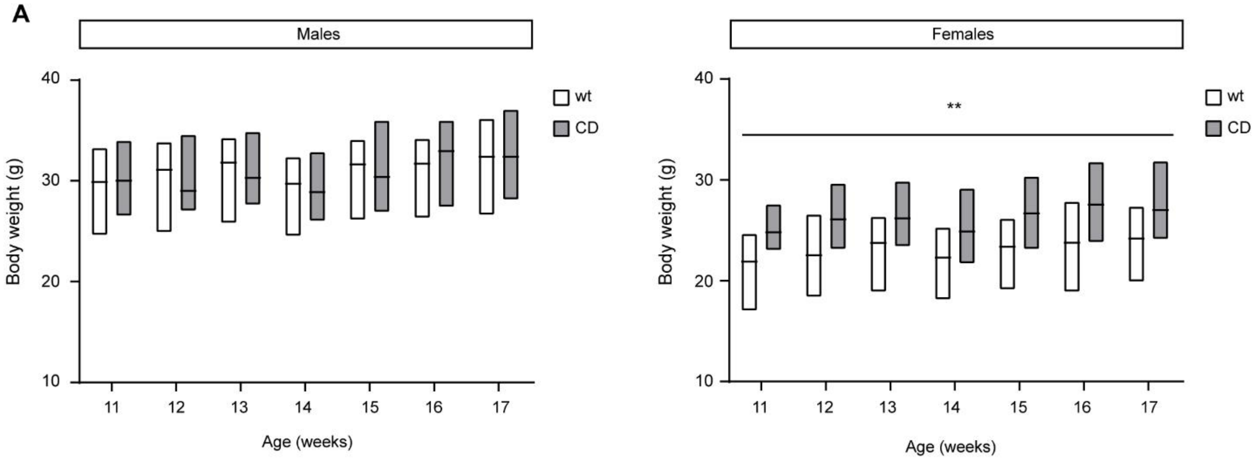

B

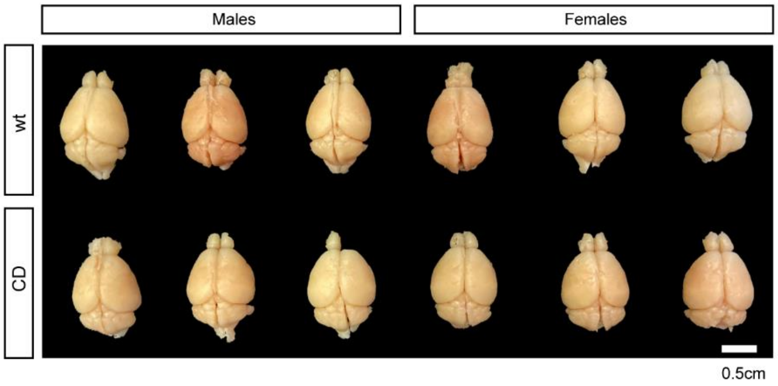

C

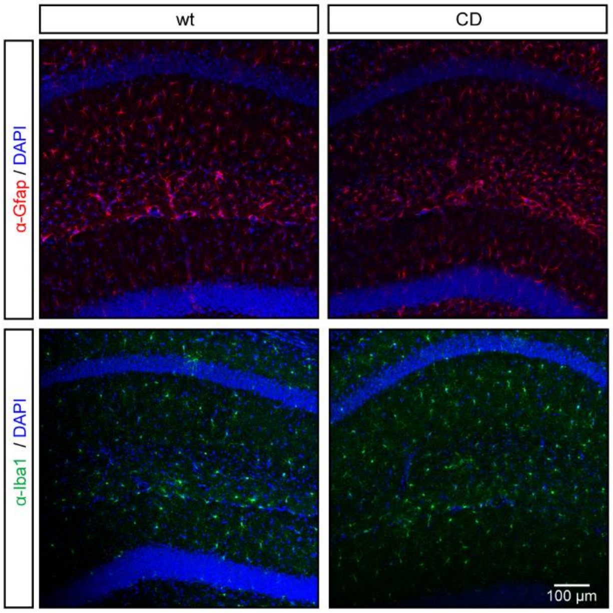

Fig. S6. Body weight and brain size of the circTulp4-deficient (CD) mouse line. (**A**) CD female mice, but not CD male mice, show increased body weight (Mixed-effects model). Bars are limited by minimum and maximum values, with a line at mean value, $^{**}P < 0.01$. (**B**) No differences between genotypes in gross brain anatomy of 12-week-old male and female mice were found. (**C**) Representative images of immunofluorescence analysis with GFAP (astrocyte marker) and IBA1 (microglia marker) antibodies in brain sections of CD and wild type mice.

Giusti, *et al.* Supplementary Figure 7

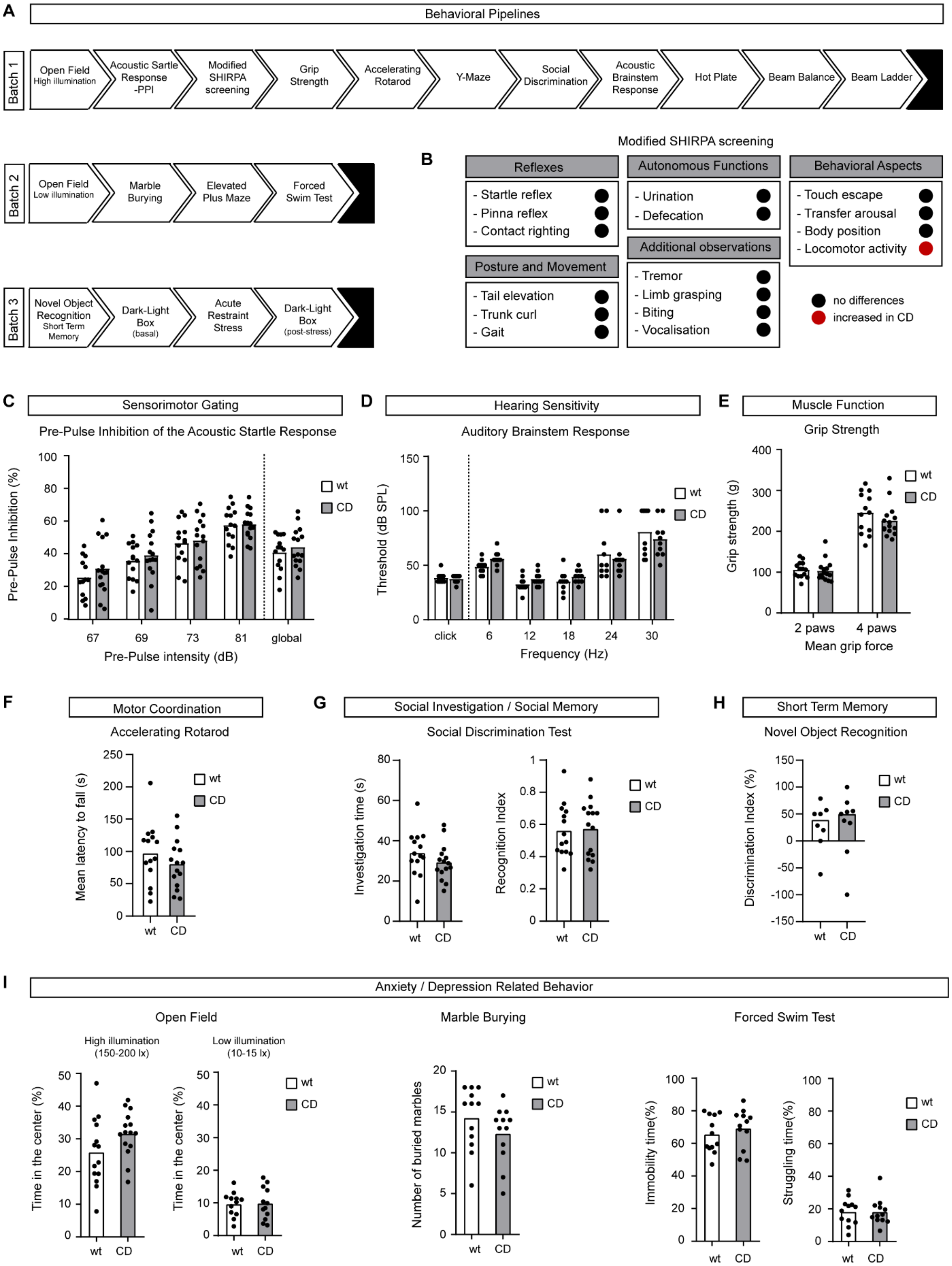

Fig. S7. Behavioral characterization of the circTulp4-deficient mouse line. (**A**) Three pipelines were used for behavioral phenotyping, each with a different batch of male mice (11-18 weeks old). Batch 1, n: 14 wt/ 15 CD; Batch 2, n: 12 wt/ 12 CD; Batch 3, n: 11 wt/ 9 CD. (**B**). The modified SHIRPA screening was used to generate a basic behavioral, physiological, and neurological profiling of CD mice. CD mice showed increased locomotor activity (details in Figure 4D). (C-I). No differences between genotypes were found in the following behavioral traits. (C) Pre-pulse inhibition of the acoustic startle response was used to estimate sensorimotor gating (Two-way RM ANOVA). (**D**) Hearing sensitivity was estimated by measuring auditory evoked potentials in a subset of mice (Mixed-effects model). (**E**) Muscle function was assessed by measuring the grip strength of the forelimbs and combined fore and hindlimbs. Each mouse was measured three times consecutively with 2 and 4 paws (linear model). (**F**) Motor coordination was assessed with the accelerating rotarod test (accelerating speed: 4 to 40rpm in 300 sec). The test consisted of three trials per mouse with 15 min intertrial intervals. Mean latencies to fall for each mouse were compared (t test). (**G**) Social investigation and social memory were assessed with the social discrimination test. The procedure consisted of two exposure trials of 4 min to an unfamiliar mouse. After a retention interval of 2 h, the test animal was re-exposed to the now familiar animal, together with an additional new, unfamiliar animal. The duration of the investigatory behavior of the test animal towards the unfamiliar animal was recorded. A social recognition index was calculated as the ratio between the time spent investigating the unfamiliar mouse to the time spent investigating both the familiar and unfamiliar mice. (**H**) An object recognition procedure was performed with one 3 min sample trial and a 3 min test trial 3h later. The experimental arena settings are identical to those described for the OF under low illumination conditions. No differences were found between genotypes in the recognition index. (**I**) Some readouts of anxiety or depression-like behavior were not affected in CD mice. *Left*: Percentage of time spent in the center of the open field under high or low illumination conditions (t test). *Middle*: Number of marbles buried during the 30 min test (t test). *Right*: Depression-like behavior was assessed with the forced-swim test. Percentages of immobility and struggling time were compared (t-test). In all cases, columns represent mean values.

Giusti, *et al.* Supplementary Figure 8

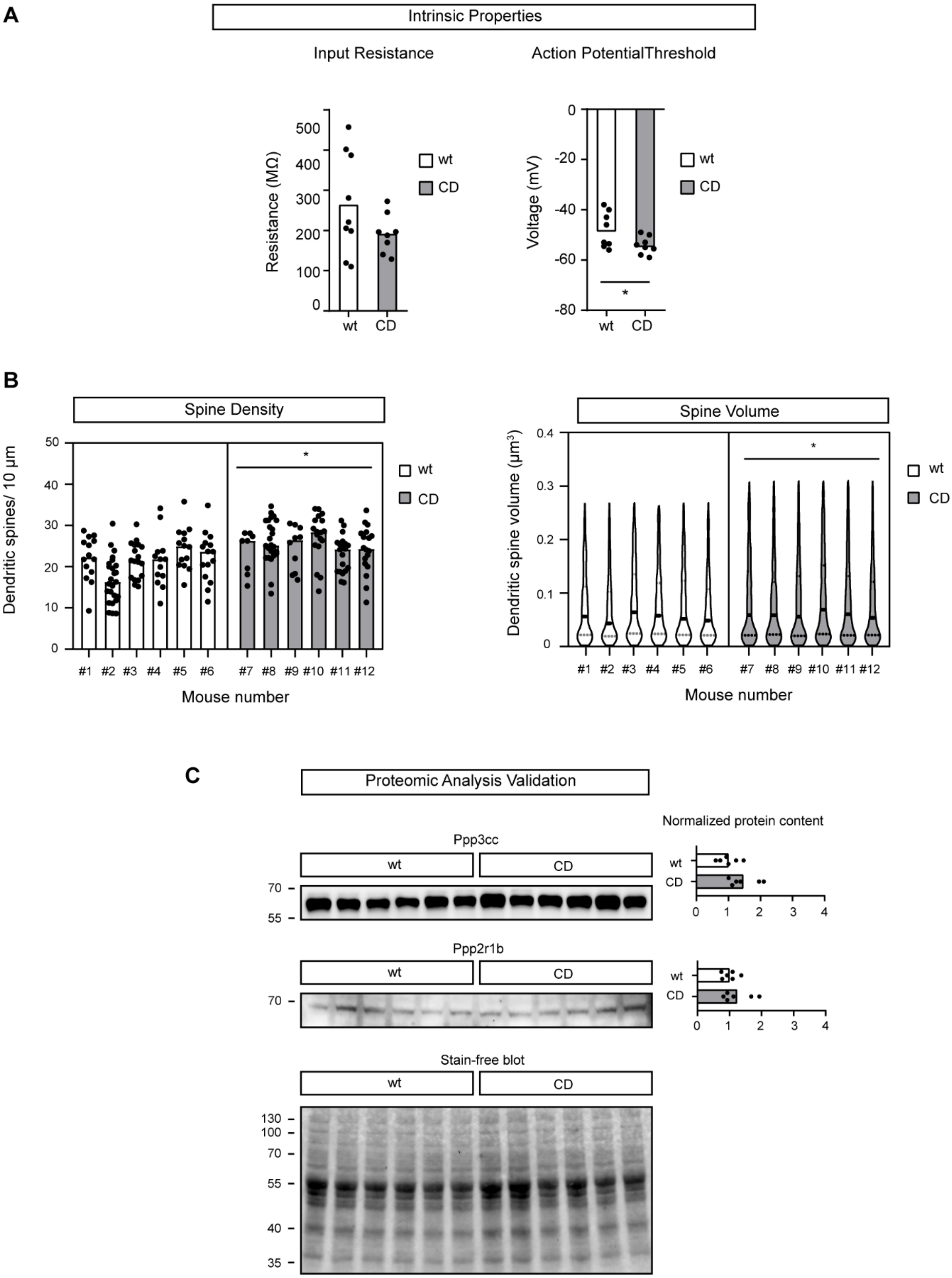

Fig. S8. Electrophysiological and morphological compensatory mechanisms in CD mice. (**A**) Intrinsic properties to current pulses. Input resistance was not affected in CD mice CA1 neurons (t test with Welch's correction, n= 9 cells from 2 mice for wt and 8 cells from 3 mice for CD mice). Action potential threshold was shifted to more negative membrane potentials in CA1 neurons of CD mice (t test, n= 8 cells from 2 mice for wt and 8 cells from 3 mice for CD mice). Columns represent mean values. $*P < 0.05$. (**B**) Morphological analysis of CA1 neurons of the circTulp4-deficient mouse line. Average spine density (left) and spine volume (right) in wild type and CD mice. In both cases, data were grouped by mouse and analyzed by a nested t test. $*P < 0.05$. (**C**) Western blot of pure synaptosomes did not corroborate the dysregulation of Ppp3cc and Ppp2r1b observed in the proteomic analysis (t test with Welch's correction, n= 6 samples/genotype).

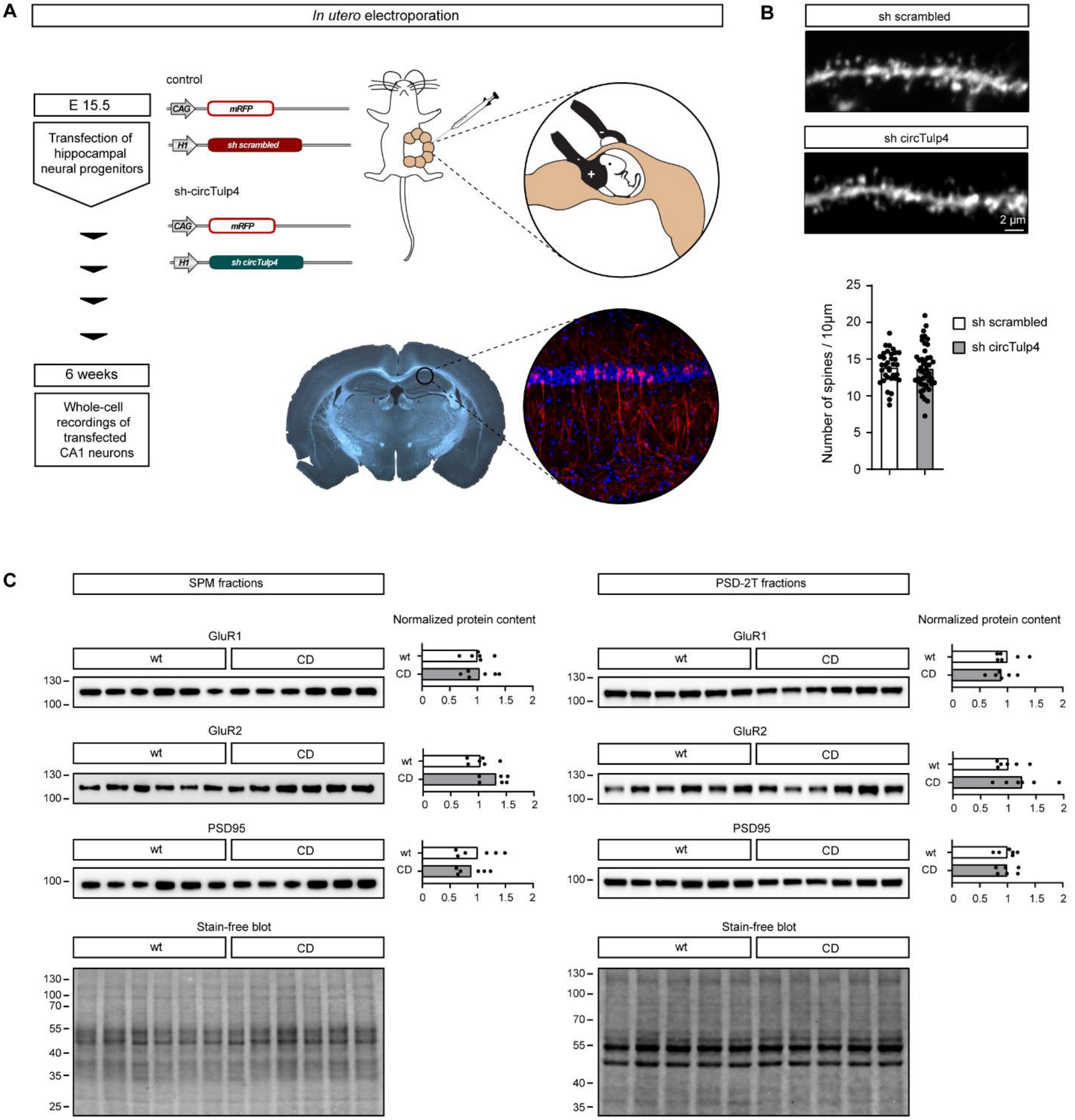

Fig. S9. Downregulation of circTulp4 in CA1 neurons of wild type mice with the *in utero* electroporation technique. (A) Schematic outline depicting the *in utero* electroporation technique. Hippocampal neural precursors of wild type CD1 embryos were in utero electroporated at embryonic day [E] 15.5 with an shRNA vector that selectively downregulates circTulp4 (sh-circTulp4). A scrambled shRNA sequence (sh-scrambled) was used in the control condition. In both conditions, mRFP was cotransfected to identify transfected neurons. Six weeks after birth, electroporated CA1 neurons were subjected to electrophysiological and morphological analysis. (B) CircTulp4 downregulation did not affect the density of dendritic spines in transfected CA1 neurons (t test, n= spine density of 34 dendritic segments for sh-scrambled and 47 for sh-circTulp4,

from 2 mice in each case). Columns represent mean values. (C) Western blot of total Synaptic Plasma Membranes (SPMs) and Postsynaptic-density anchored protein fraction (PSD-2T) demonstrate that there is no difference between genotypes in the anchorage of GluR1 and GluR2 on the post-synaptic density (t test with Welch's correction, n= 6 samples/genotype). In all cases, columns represent mean values.of wild type CD1 embryos were in utero electroporated at embryonic day [E] 15.5 with an shRNA vector that selectively downregulates circTulp4 (sh-circTulp4). A scrambled shRNA sequence (sh-scrambled) was used in the control condition. In both conditions, mRFP was cotransfected to identify transfected neurons. Six weeks after birth, electroporated CA1 neurons were subjected to electrophysiological and morphological analysis. (**B**) CircTulp4 downregulation did not affect the density of dendritic spines in transfected CA1 neurons (t test, n= spine density of 34 dendritic segments for sh-scrambled and 47 for sh-circTulp4, from 2 mice in each case). Columns represent mean values. (**C**) Western blot of total Synaptic Plasma Membranes (SPMs) and Postsynaptic-density anchored protein fraction (PSD-2T) demonstrate that there is no difference between genotypes in the anchorage of GluR1 and GluR2 on the post-synaptic density (t test with Welch's correction, n= 6 samples/genotype). In all cases, columns represent mean values.

Giusti, *et al.* Supplementary Figure 10

A

Predicted miRNA binding sites in circTulp4		
miRNA Name	Target score	Seed location in circTulp4 sequence
mmu-miR-1b-5p	83	1692
mmu-miR-879-3p	82	140
mmu-miR-7050-3p	82	1335, 1350
mmu-miR-26b-5p	81	1213, 1267, 1438
mmu-miR-26a-5p	81	1213, 1267, 1438
mmu-miR-6985-5p	79	1655
mmu-miR-671-3p	79	362
mmu-miR-7078-3p	76	1688
mmu-miR-7015-5p	75	302
mmu-miR-190b-5p	75	73
mmu-miR-190a-5p	75	73
mmu-miR-7229-3p	75	788, 1459
mmu-miR-26a-1-3p	70	320, 1225

B

Gm36251	
miRNA Name	Target score
mmu-miR-6413	82
mmu-miR-6410	82
mmu-miR-6369	82
mmu-miR-6361	82
mmu-miR-5124b	82
mmu-miR-24-3p	82
mmu-miR-3074-2-3p	79
mmu-miR-6335	76
mmu-miR-671-5p	75
mmu-miR-6359	74
mmu-miR-6407	72
mmu-miR-7010-5p	71
mmu-miR-3079-3p	71
mmu-miR-1251-3p	70
mmu-miR-6945-5p	70

Gm2694	
miRNA Name	Target score
mmu-miR-5101	91
mmu-miR-5120	91
mmu-miR-7116-3p	91
mmu-miR-139-3p	88
mmu-miR-6951-3p	86
mmu-miR-7094-3p	81
mmu-miR-6962-5p	80
mmu-miR-466h-5p	77
mmu-miR-6931-5p	76
mmu-miR-669m-5p	75
mmu-miR-466m-5p	75
mmu-miR-466j	75
mmu-miR-7688-3p	72
mmu-miR-7218-5p	72
mmu-miR-12195-5p	72
mmu-miR-12188-3p	70
mmu-miR-466k	70
mmu-miR-466d-5p	70

Ccn3	
miRNA Name	Target score
mmu-miR-7030-3p	94
mmu-miR-3073a-3p	94
mmu-miR-7078-3p	91
mmu-miR-489-5p	86
mmu-miR-3075-5p	81
mmu-miR-1a-2-5p	79
mmu-miR-1a-1-5p	79
mmu-miR-344h-3p	78
mmu-miR-7084-3p	76
mmu-miR-421-5p	74
mmu-miR-6949-5p	73
mmu-miR-6900-3p	72
mmu-miR-30e-3p	71
mmu-miR-30d-3p	71
mmu-miR-30a-3p	71
mmu-miR-6350	70
mmu-miR-6346	70
mmu-miR-6996-5p	70

H19	
miRNA Name	Target score
mmu-miR-6931-5p	86
mmu-miR-721	84
mmu-miR-130b-3p	84
mmu-miR-130a-3p	84
mmu-miR-6389	80
mmu-miR-6341	80
mmu-miR-301b-3p	80
mmu-miR-301a-3p	80
mmu-miR-130c	80
mmu-miR-7078-5p	78
mmu-miR-698-5p	78
mmu-miR-6942-5p	75
mmu-miR-6916-5p	75
mmu-miR-6954-5p	75
mmu-miR-6922-3p	75
mmu-miR-12184-3p	74
mmu-miR-709	72
mmu-miR-743b-3p	70
mmu-miR-743a-3p	70
mmu-miR-486b-3p	70
mmu-miR-486a-3p	70
mmu-miR-326-5p	70
mmu-miR-6988-5p	70
mmu-miR-6903-5p	70

C

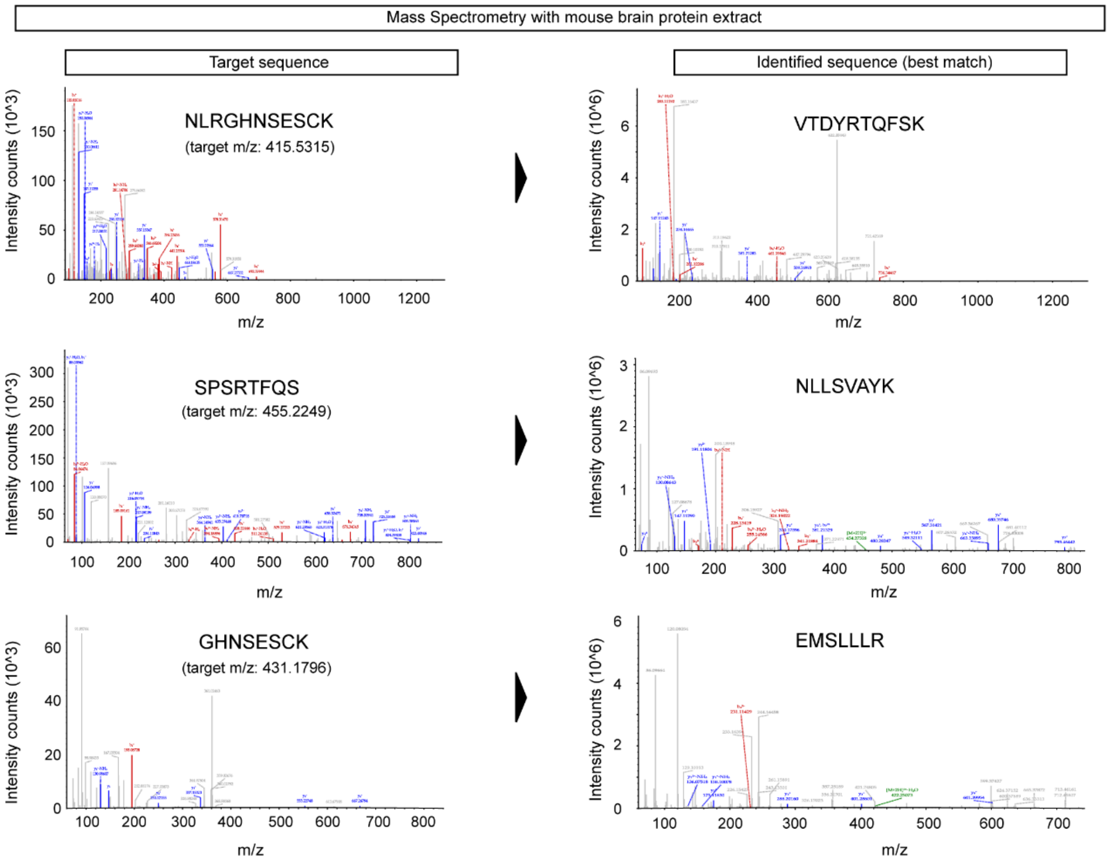

Fig. S10. Bioinformatic predictions of miRNA binding sites and MS/MS spectra of the mass spectrometry analyses. (**A**) Bioinformatically predicted miRNA binding sites in the circTulp4 sequence. (**B**) Bioinformatically predicted miRNA binding sites in mRNA targets, which are dysregulated in CD mice brains. In both cases, only target sites with a score of 70 or higher are shown. (**C**) The MS/MS spectra corresponding to mass spectrometry analyses reveal that brain samples do not contain the theoretical peptide circ-specificTulp4. First, the synthetic theoretical peptides (NLRGHNSESCK, GHNSESCK, SPSRTFQS, 1pmol each) were analyzed to collect MS/MS reference spectra. Then, a mouse brain protein digest (1 µg) was injected into the mass

spectrometer using Parallel Reaction Monitoring (PRM) mode with an inclusion list for the theoretical peptides following a full MS scan. No exact matches were found.

Table S1. Oligonucleotides used in this study

Oligonucleotide name	Use		Sequence (5' to 3')
circTulp4	qPCR	fw	TCACTGTCGCAGAGATAGGAGT
		rv	GGCACTTGATATGTTTGTTTTCC
linearTulp4	qPCR	fw	GAAGCCACACAGGTGTACCA
		rv	TCCGTCCAAACTGCATCAC
Tulp4-204	qPCR	fw	GTCTGGTTGATGCAGCTGAG
		rv	GCCAAGCATCTTCACGTTG
Tulp4-205	qPCR	fw	TGACAAGCTCGCCTGTCACT
		rv	GCTCCACGTGAAATCACTCACC
Tulp4-210	qPCR	fw	ATCCACCTACTCCAACAAGGCC
		rv	TTCCCGGTTAATTCAGGAGCCA
Tulp4-212	qPCR	fw	GAGAAGGAGGCGGAGAGG
		rv	TGATATGTTTGTTTTCCCGGTTA
Gabra1	qPCR	fw	AAAAGTCGGGGTCTCTCTGAC
		rv	CAGTCGGTCCAAAATTCTTGTGA
Slc12a5/Kcc2	qPCR	fw	GGGCAGAGAGTACGATGGC
		rv	TGGGGTAGGTTGGTGTAGTTG
Synaptotagmin1	qPCR	fw	GGGAGGCACATCTGATCCATA
		rv	TTCCGGTGGACTTTTGTCTCA
Slc17a7/Vglut1	qPCR	fw	GGTGGAGGGGGTCACATAC
		rv	AGATCCCGAAGCTGCCATAGA
Gfap	qPCR	fw	GGGGCAAAAGCACCAAAGAAG
		rv	GGGACAACTTGTATTGTGAGCC
Aldh1L1	qPCR	fw	CAGGAGGTTTACTGCCAGCTA
		rv	CACGTTGAGTTCTGCACCCA
Aquaporin4	qPCR	fw	CTTTCTGGAAGGCAGTCTCAG
		rv	CCACACCGAGCAAAACAAAGAT
Acsbg1	qPCR	fw	ATGCCACGCGGTTCTGAAG
		rv	GAGCTGGTTTGCGAGTTGTCT
Kif5c	qPCR	fw	TGTCCAAGACCAACTTGGCAGTTC
		rv	TGTGACTCCTAGAGCTGTGTTCGT
Bassoon	qPCR	fw	AGCCAGCCAAACTTCAACACCTG
		rv	AGGCGCAGTGGTCATGTCCATT
Adcy1	qPCR	fw	GGTGTCCTCCTTGCCAAAGATGA
		rv	CAGCCGTAGTCTGACATCCACTT
Npas4	qPCR	fw	CTGCATCTACACTCGCAAGG
		rv	GCCACAATGTCTTCAAGCTCT
cfos	qPCR	fw	ATGGGCTCTCCTGTCAACACAC
		rv	ATGGCTGTCACCGTGGGGATAAAG

Arc	qPCR	fw	TACCGTTAGCCCCTATGCCATC
		rv	TGATATTGCTGAGCCTCAACTG
RPL19	qPCR	fw	GCATCCTCATGGAGCACAT
		rv	CTGGTCAGCCAGGAGCTT
TATAbinding protein	qPCR	fw	GTGATGTGAAGTTCCCCATAAGG
		rv	CTACTGAACTGCTGGTGGGTCA
CD mouse line	genotyping	fw	CCGGCAGGAACAAGGGATAG
		rv	GGTGCTCCAGCTGATGCTTA
Thy1EGFP mouse line	genotyping	fw	TCTGAGTGGCAAAGGACCTTAGG
		rv	GTCCTCCTTGAAGTCGATGC
sh scrambled	shRNA		GCGCGCTTTGTAGGATTCG
sh circTulp4	shRNA		CAGTGAGAGTTGTAAGAGT
sh linear Tulp4	shRNA		GAGCTGATGAACCAAAGCC
gRNA_SA_circTulp4#1	For cloning into Pbs_U6_chimaeric RNA vector		AGAGTTGTAAGAGTCCATCC
gRNA_SA_circTulp4#2	For cloning into Pbs_U6_chimaeric RNA vector		GTAAAACAACGACAAAAAAG
ssODN_SA_circTulp4	Donor template for deleting the splicing acceptor site from circTulp4		ACACACACATGCATATATACATATATATATACACACACACACATATCCTCTTTTTTGTCGTTGTTTTACTCAGTTGTAAGAGTCCATCCAGTACCTTCCAGTCATGAATAATCTGATGGCTCCTGAATTAACCGGGAAAA
gRNA_Met_Tulp4	For cloning into Pbs_U6_chimaeric RNA vector		TGTATGCAGCAGTGGAACAT

Table S2. Antibodies used in this study

Antibody	Company, catalog number	Use
α-TULP4	Aviva Systems Biology, #ARP39410_P050	WB
α-SynapsinI	SySy, #106 011	WB
α-PSD95	NeuroMab, #75-028, clone K28/43	WB
α-β-Actin	CST, #4967	WB
α-HA	BioLegend (previously Covance, # MMS-101P)	WB
α-GluR1	Millipore, MAB2263, clon RH95	WB
α-GluR2	NeuroMab, #75-002, clon L21/32	WB
α-GluR1, phosphoSer 831	Millipore, AB5847	WB
α-GluR1, phosphoSer 845	Millipore, AB5849	WB
α-PPP3CC	GeneTex, GTX55755	WB
α-PPP2R1B [EPR10158]	Abcam, #ab154815	WB
α-mouse-IgG HRP	CST, #7076	WB
α-rabbit-IgG HRP	CST, #7074	WB
α-GAPDH (D16H11)-HRP	CST, #8884	WB
α-MAP2	Abcam, #ab32454	IF
α-GFAP	Dako Cytomation, #00005193	IF
α-Iba1	Wako, #019-19741	IF
Alexa Fluor 488 goat α-rabbit IgG	Invitrogen, #A11034	IF
Alexa Fluor 594 goat α-rabbit IgG	Invitrogen, #A11037	IF
Alexa Fluor 647 goat α-rabbit IgG	Invitrogen, #A21244	IF
Alexa Fluor 488 goat α-mouse IgG	Invitrogen, #A11029	IF
Alexa Fluor 594 goat α-mouse IgG	Invitrogen, #A11032	IF
Alexa Fluor 647 goat α-mouse IgG	Invitrogen, #A21236	IF

Data S1 (Excel file): RCMs identified by BLASTn results from flanking introns.

Data S2 (Excel file): Label-free quantification of proteins in pure synaptosomal fractions from circTulp4-deficient (CD) and wild type (wt) mice.
